# Non-Canonical CRL4A/4B^CDT2^ Interacts with RAD18 to Modulate Post Replication Repair and Cell Survival

**DOI:** 10.1371/journal.pone.0060000

**Published:** 2013-03-29

**Authors:** Sarah Sertic, Claudio Evolvi, Emanuela Tumini, Paolo Plevani, Marco Muzi-Falconi, Giuseppe Rotondo

**Affiliations:** Dipartimento di Bioscienze, Università degli Studi di Milano, Milano, Italy; University Medical Center Hamburg-Eppendorf, Germany

## Abstract

The Cullin-4^CDT2^ E3 ubiquitin ligase plays an essential role in DNA replication origin licensing directing degradation of several licensing factors at the G1/S transition in order to prevent DNA re-replication. Recently a RAD18-independent role of Cullin-4^CDT2^ in PCNA monoubiquitylation has been proposed. In an effort to better understand the function of Cullin-4^CDT2^ E3 ubiquitin ligase in mammalian Post-Replication Repair during an unperturbed S-phase, we show that down-regulation of Cullin-4^CDT2^ leads to two distinguishable independent phenotypes in human cells that unveil at least two independent roles of Cullin-4^CDT2^ in S-phase. Apart from the re-replication preventing activity, we identified a non-canonical Cullin-4^CDT2^ complex, containing both CUL4A and CUL4B, associated to the COP9 signalosome, that controls a RAD18-dependent damage avoidance pathway essential during an unperturbed S-phase. Indeed, we show that the non-canonical Cullin-4A/4B^CDT2^ complex binds to RAD18 and it is required to modulate RAD18 protein levels onto chromatin and the consequent dynamics of PCNA monoubiquitylation during a normal S-phase. This function prevents replication stress, ATR hyper-signaling and, ultimately, apoptosis. A very similar PRR regulatory mechanism has been recently described for Spartan. Our findings uncover a finely regulated process in mammalian cells involving Post-Replication Repair factors, COP9 signalosome and a non-canonical Cullin4-based E3 ligase which is essential to tolerate spontaneous damage and for cell survival during physiological DNA replication.

## Introduction

The maintenance of genome integrity is fundamental for cell survival and controlled cell growth. Indeed, most cancer cells exhibit genome instability, often arising from DNA replication defects and faulty repair events [1], [2].

Cullin-RING Ub ligases (CRLs) are the largest family of E3 ubiquitin ligases and they play a fundamental role in a variety of cellular processes. Each CRL consists of a common core complex, containing the Cullin scaffold subunit, the Rbx1 RING subunit and an adaptor protein, that assembles to a substrate receptor subunit that provides specificity to each CRL [3]. Increasing evidence shows that variants of this canonical architecture exist, extending the CRLs family complexity and functionality (for a review see [4]). CRLs are divided in sub-families according to the specific Cullin in the core complex. Members of the CRL4s family, which contains the Cullin-4A or the Cullin-4B scaffold protein, are important in the DNA Damage Response (DDR) [5], [6]. CRL4A and CRL4B have been reported to have some redundant functions in DDR [7], [8]. However, CUL4B plays also roles in the DDR that are not shared with CUL4A [9]. Similarly, degradation of p27 and p53 has been shown to depend solely on CUL4A [10], [11]. Further investigations are required to advance our knowledge on the CUL4A and CUL4B relationship in DDR.

The COP9 signalosome (CSN) is an eight subunits protein complex acting as a platform for CRL complexes and protein kinases [12]. CSN has intrinsic de-neddylation and deubiquitylation enzymatic activities, which regulate CRLs biogenesis and function (for a review see [13], [14]). Similarly to CRL4s, CSN has been associated with several aspects of DDR [15], [16]. Particularly, UV irradiation causes CDT1 degradation due to CSN-mediated CRL4^CDT2^ activation [7]. However, a possible involvement of CSN in modulating CRL4^CDT2^ -dependent degradation of CDT1 at the G_1_/S transition, preventing DNA re-replication in an unperturbed cell cycle, has not been described [10].

DNA replication must occur only once per cell cycle. This is achieved by restricting origin firing to once per S-phase. Re-initiation from even one single origin within the same cell cycle may cause genome instability [17]; consequently, re-replication is one of the most common early events in tumorigenesis [18], [19], [20]. Two main mechanisms contribute to preventing origin to fire more than once per each cycle. One impedes re-loading of the MCM2-7 helicase onto a G_1_-assembled post-replication complex, preventing re-formation of an active pre-replication complex after a specific origin has fired. This is achieved by coupling spatially and temporally the selective ubiquitylation and degradation of licensing factors by CRL4^CDT2^ (particularly CDT1, p21 and SET8) to the loading of PCNA on chromatin as the origin fires (for a review see [21]). Indeed, CRL4^CDT2^ is able to mark for destruction only the CDT1, p21 and SET8 population bound to PCNA on chromatin [3], [10], [22], [23], [24], [25], [26], [27], [28]. Due to this function in preventing DNA re-replication, CRL4^CDT2^ deregulation leads to ATM-dependent checkpoint activation and correlates with tumorigenesis [29], [30].

A second mechanism avoids the reassembly of a new pre-replication complex at an origin during S or G_2_. This is achieved thanks to Skp2, a Cullin1-based ubiquitin E3 ligase, that keeps CDT1 and SET8 protein levels very low in S and G_2_ despite CDT1 and SET8 being expressed constantly throughout the cell cycle [31], [32].

During S-phase, DNA lesions are mostly tolerated through Post-Replication Repair (PRR) mechanisms that allow lesions bypass and completion of DNA replication (for a review see [33]). Studies in yeast have indicated that PRR is essential for DNA replication following exogenous DNA damage, but not during normal replication [34]. PRR includes error-free recombination mechanisms and error-prone processes mostly employing multiple translesion DNA polymerases (for a review see [35], [36]). PCNA ubiquitylation (mono- or poly- ubiquitylation) acts as a molecular switch to control the choice between these two PRR sub-pathways [34]. RAD18 and RAD5, are the two major players in the yeast PRR pathway, and code for the E3 ubiquitin ligases required for PCNA monoubiquitylation and polyubiquitylation, respectively.

In mammalian cells the picture is more complex. A RAD18 homolog and two RAD5 homologs, HLTF and SHPRH, have been identified in human cells (for a review see [37], [38]). Intriguingly, in response to different DNA damaging agents cells differentially employ HLTF and SHPRH together with RAD18 to modify PCNA and recruit lesion-specific translesion polymerases, promoting error-free TLS [39]. A feed-forward loop, which amplifies the chromatin-bound monoubiquitylated PCNA population and consequently translesion DNA synthesis, has been described very recently. Spartan, a protein that binds monoubiquitylated PCNA, has been shown to be critical for obtaining a large population of monoubiquitylated PCNA [40]. The molecular details of such amplification loop have not been fully understood yet.

A further layer of complexity is added by the observation that CRL4^CDT2^ ubiquitin ligase can monoubiquitylate PCNA *in vitro* and *in vivo*, functionally synergizing with RAD18 [41], but the crosstalk between CRL4^CDT2^ and PRR factors are still unclear. Indeed, how loss of the PRR regulatory function of CRL4^CDT2^ affects unperturbed S-phase progression in normal or cancer human cells needs to be clarified.

Here we report the existence of a non-canonical CSN-CRL4A/4B^CDT2^ complex, and its genetic and biochemical interactions with HLTF, SHPRH, RAD18 and PCNA. Such complex regulates PCNA ubiquitylation, modulating RAD18 recruitment to chromatin, similarly to what observed with Spartan; this helps cells to cope with DNA replication stress during a normal S-phase and to avoid apoptosis. Furthermore, our findings indicate that PRR is critical for survival in undamaged human cells, identifying PRR components as possible pharmacological targets to induce apoptosis in cancer cells

## Results

### Depletion of CRL4^CDT2^ or CSN activates the DDR in S-phase

Depletion of CRL4^CDT2^ subunits causes activation of markers linked to either replication stress or DNA damage, along with a cell cycle arrest in G_2_. This phenotype has been shown to depend, at least in part, on the DNA re-replication caused by failure to degrade replication origin licensing proteins [3], [25], [26], [42]. The available data do not exclude, though, the possibility that DDR activation may be the composite result of deregulating more independent mechanisms controlled by CRL4^CDT2^ in S-phase.

**Figure 1:**
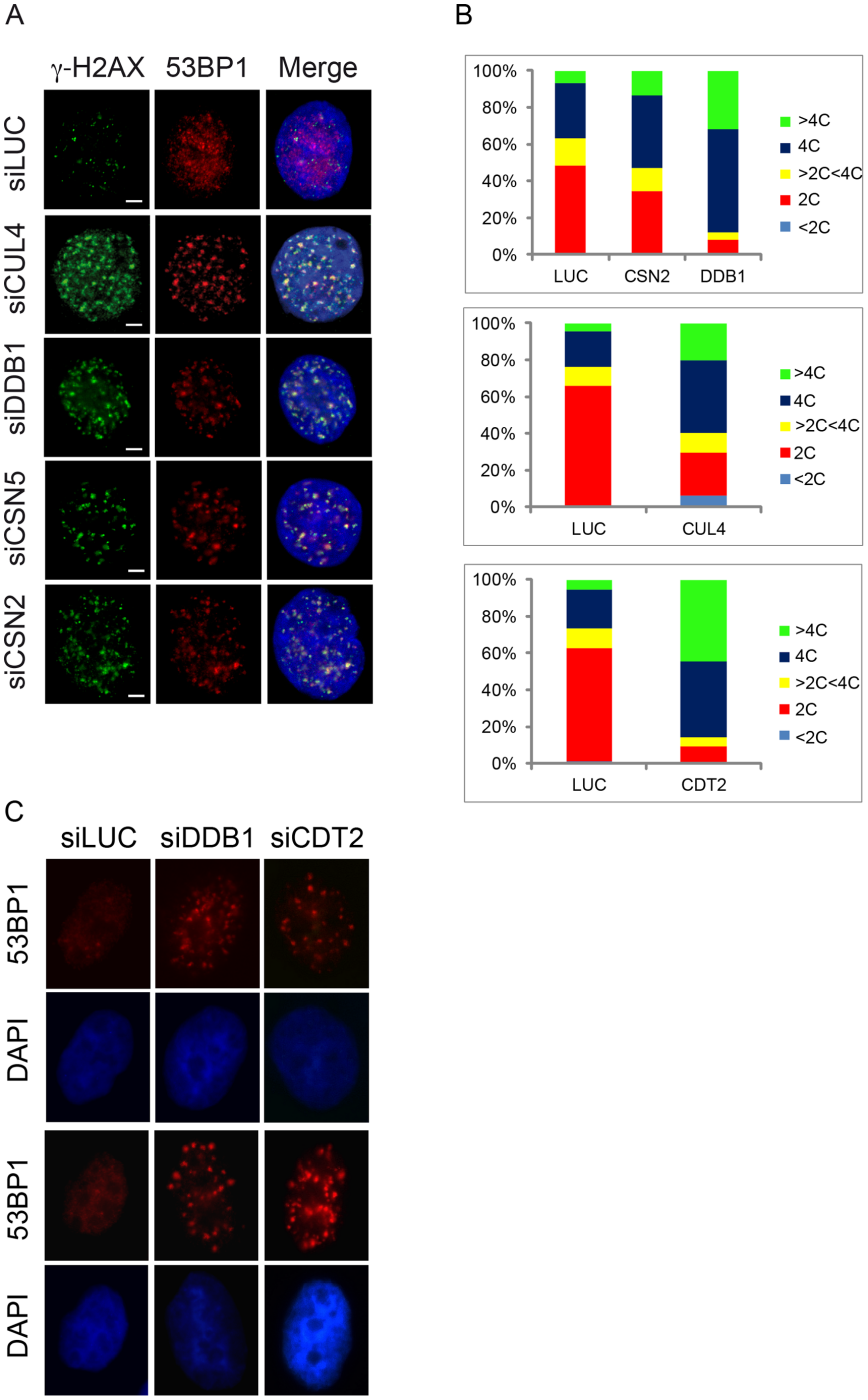
CSN-CRL4^CDT2^-depletion causes DDR activation and DNA re-replication. Exponentially growing HeLa cells depleted for the indicated proteins were harvested for further analysis. (A) Cells were fixed and stained with antibodies to H2AX phospho-S139 (γH2AX) and 53BP1; the nucleus was counterstained with DAPI. A fluorescent image of a representative nucleus is shown. (B) Cell cycle distribution was analyzed by flow cytometry monitoring the DNA content. For each representative FACS diagram, the percentage of cells in the various cell cycle phases was calculated and displayed as relative value chart. (C) S-phase synchronized HeLa cells depleted for the indicated proteins were fixed and stained with 53BP1 antibodies; the nucleus was counterstained with DAPI. Fluorescent images of two representative nuclei for each sample are shown.

To investigate possible new roles of CRL4^CDT2^ during normal S-phase and their influence on the DDR, we depleted CRL4^CDT2^ subunits in dividing cells. Because most of the mammalian-based work on CUL4 E3 ligases rarely makes a functional distinction between CUL4A and CUL4B, we down-regulated concomitantly CUL4A and CUL4B isoforms (indicated as CUL4) using siRNA (small interfering RNA). To support the data and to validate the target specificity of our siRNAs, we down-regulated DDB1, another CRL4 core complex subunit. Furthermore, since in many cellular processes CSN is associated to and regulates the function of members of the CRL4s family, we investigated a possible involvement of CSN in modulating the DDR during a normal S-phase. DDR activation was monitored by immunofluorescence (IF) staining Ser-139-phosphorylated histone H2AX (γH2AX) and 53BP1 nuclear foci. Exponentially growing HeLa cells transfected with a control siRNA against luciferase (siLUC) show a very weak punctuate γH2AX staining while evident 53BP1 nuclear foci are absent. On the contrary, cells depleted of either CRL4^CDT2^ subunits (CUL4 or DDB1) or CSN subunits (CSN2 or CSN5) exhibit a strong γH2AX nuclear signal that is mostly organized in discrete foci, and 53BP1 foci formation (Figure 1A). Cells positive for 53BP1 were also positive for γH2AX and vice versa, and most of 53BP1 foci colocalize with γH2AX foci (Figure 1A). The phenotypes show different penetrance (number of cells positive for 53BP1 foci) and severity (number of 53BP1 foci per cell) depending on depleted protein (Figure S1A). To confirm these data, the total levels of γH2AX were also evaluated by immunoblotting on the same samples from Figure 1A. Depletion of any of the indicated proteins strongly induces H2AX phosphorylation (Figure S1B).

The cell cycle distribution of exponentially growing HeLa cells depleted for CRL4 core and CSN subunits, was estimated by FACS analysis and compared to control cells (Figure 1B and Figure S2A). In agreement with previous reports [15], our data indicate that both CRL4 core complex and CSN depletion activate the DDR markers and delay cell cycle progression, causing an increase in the fraction of population with 4C DNA content, indicative of G_2_-M cells and to the accumulation of cells with greater than 4C DNA content (>4C in Figure 1B), which is due to DNA re-replication. We observed a similar phenotype in CDT2-depleted HeLa cells (Figure 1B and Figure S2A), suggesting that the observed phenotypes are specifically due to downregulation of the CRL4^CDT2^ ubiquitin ligase complex. To discriminate whether cell populations that accumulate with 4C and >4C DNA contents were in G_2_ or mitosis, DDB1-depleted cells were immunostained for Ser10 phosphorylation of histone H3, a mitotic marker. DDB1 depletion does not significantly increase the percentage of mitotic cells (Figure S2B). Therefore we assume that depletion of CRL4^CDT2^ and CSN likely causes cells with 4C and >4C DNA content to accumulate in G_2_. Similar results were confirmed in U2OS cells (Figure S3A and S3B).

In order to verify that DDR activation was due to dysfunctional replication, HeLa cells synchronized in S-phase (as checked by FACS analysis shown in Figure S2C) and depleted of either DDB1 or CDT2 show activation of the 53BP1 DDR marker by IF (Figure 1C) similarly to what found in exponentially growing cells depleted of the same proteins (Figure 1A).

### CSN-CRL4^CDT2^ plays both CDT1-dependent and CDT1-independent functions in S-phase

During S-phase, CRL4^CDT2^ regulates origin licensing and controls translesion DNA synthesis, regulating PCNA ubiquitylation and DNA polymerase η stability [41], [43]. We investigated the contribution of these diverse functions to the activation of the DDR in CRL4^CDT2^ depleted cells.

**Figure 2:**
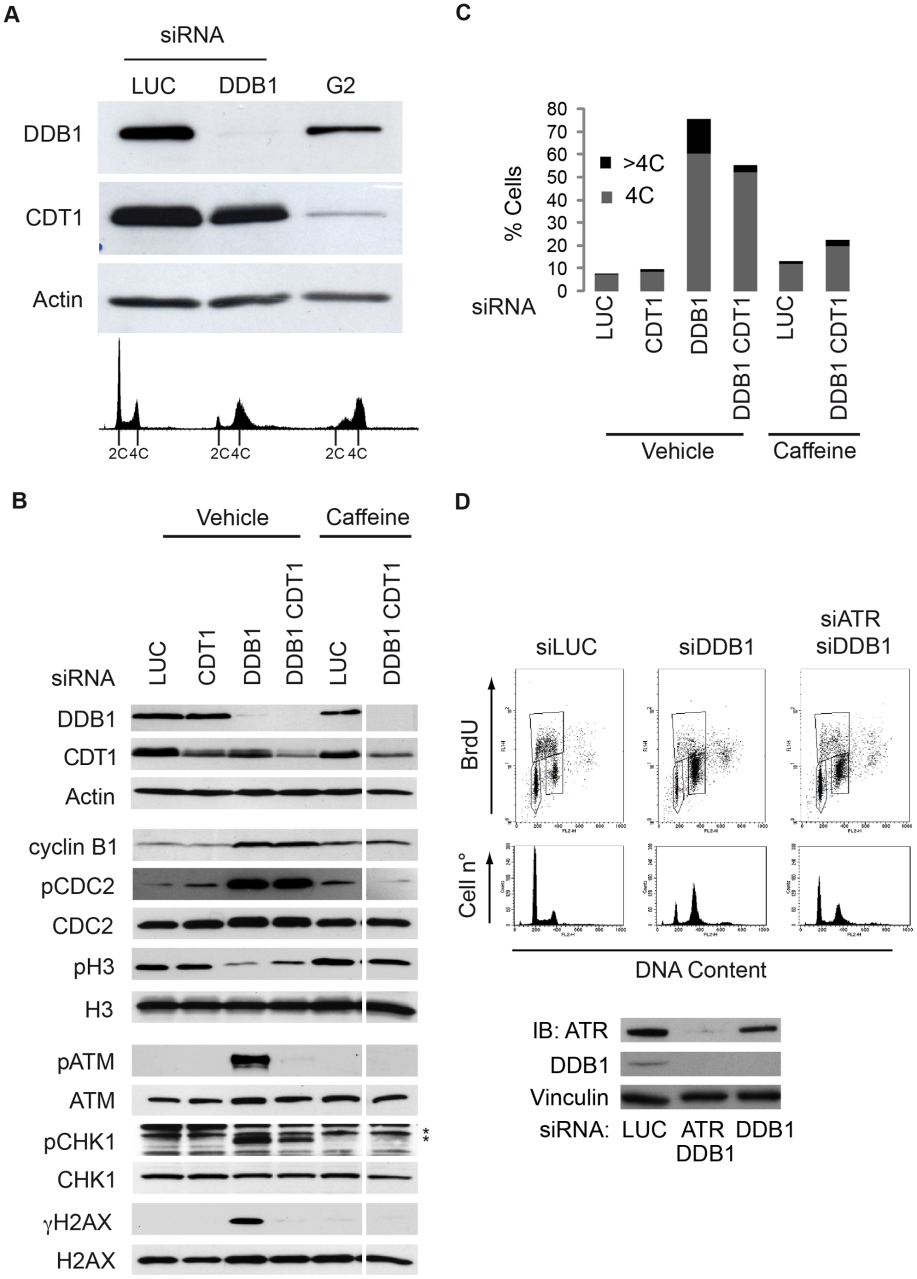
CRL4^CDT2^ has a CDT1-independent function during S-phase. (A) HeLa cells were harvested 48 hrs after the last transfection cycle with control (siLUC) or DDB1 (siDDB1) siRNAs. For G_2_ control, aphidicolin was used to block HeLa cells at the G_1_/S border. Cells were release from the aphidicolin block and harvested after 6 hours. Each cells sample was divided by two and analyzed both by by immunoblotting with the indicated antibody (top panel) and by FACS (lower panel). (B) HeLa cells were transfected with the indicated siRNAs (± caffeine) and harvested for further analysis. Total protein extracts were resolved by SDS-PAGE and immunoblotted with the indicated antibodies. * indicates not specific bands. (C) Cell cycle distribution was analyzed by flow cytometry monitoring BrdU incorporation and DNA content in Figure S4A. 4C and >4C cell populations were calculated as percentage over the total and graphically represented as bars. (D) Cell cycle distribution of HeLa cells transfected with either siATR or siDDB1 or both were analyzed by BrdU incorporation and DNA content using flow cytometry. ATM and DDB1 depletions were assessed by western blot.

As shown in Figure 1, DDB1-depleted cells recapitulate the phenotypes due to inactivation of CSN-CRL4^CDT2^. Moreover, CDT1 protein levels are high in G_1_ phase and become reduced in S and G_2_ phase. Inappropriate origin licensing following CRL4^CDT2^ inactivation depends on the failure to degrade CDT1 in S- and G_2_-phase, and to the consequent increase in CDT1 level in S- and G_2_-phase (for a review see [21]). Indeed, DDB1-depletion, despite causing >80% cells to accumulate in G_2_ (Figure 2A, see FACS profiles at the bottom), shows a strong increase in CDT1 protein, much greater than the one observed in control cells that proceed synchronously through G_2_ with an intact CRL4^CDT2^ (Figure 2A; compare DDB1 and G_2_ samples). Nevertheless, CDT1 protein level in DDB1-depleted cells appears lower compared to the steady-state level of CDT1 into logarithmically growing control cells (Figure 2A; compare LUC and DDB1 samples). Indeed, this may be explained by the fact that logarithmically growing control HeLa cells show >85% cells in G_1_ phase where CDT1 levels are high and they are not under control of the CRL4^CDT2^ activity. (Figure 2A, see FACS profiles at the bottom). To analyze CDT1-independent CRL4^CDT2^ functions in S-phase, we employed a DDB1- and CDT1- co-depletion protocol that does not eliminate CDT1, but prevents its increase in S- and G_2_-phase linked to DDB1 depletion. (Figure 2B). HeLa cells were depleted for either DDB1 or CDT1 or both. Critical protein factors were analyzed by immunoblot (Figure 2B). The distribution of cells along the cell cycle in each depleted cell population was determined by measuring BrdU incorporation and DNA content by FACS analysis (Figure S4A). Relative values of cell sub-populations containing 4C (G_2_ cells) and >4C (G_2_ cells with re-replicated DNA) DNA content in Figure S4A were calculated and represented as a vertical bar graph shown in Figure 2C. As described above, DDB1-depletion causes cells to accumulate with 4C and >4CDNA content (Figure 2C, Bar DDB1). Interestingly, concomitant CDT1 and DDB1 depletion decreases the re-replication phenotype in HeLa and U2OS cells (>4C cells in Figure 2C and Figure S7A). This result indicates that the >4C phenotype observed after depletion of CRL4^CDT2^ is likely due to deregulation of CDT1. On the other hand the G_2_-arrested cell population with a 4C DNA content, which seem not to be substantially affected by preventing CDT1 accumulation in DDB1 depleted cells, highlights origin licensing-independent functions of CRL4^CDT2^ (4C cells in Figure 2C and Figure S7A).

DDB1-depleted cells exhibit higher levels of B1 cyclin, a G_2_/M specific marker; the increased Thr14 CDC2 phosphorylation (pCDC2) together with the concomitant decrease in Ser10 histone H3 phosphorylation (pH3) (Figure 2B), indicate that DDB1-depleted cells are arrested in G_2_, confirming the results shown in Figure 1. As we have described above (Figure 2A), G_2_-arrested cells are normally expected to have extremely low CDT1 levels. However, DDB1-depleted cells, despite being mostly in G_2_ (Figure 2C), show a strong CDT1 signal (Figure 2B). Similarly, cells depleted of either CUL4A, or CUL4B or CSN show an increase in CDT1 protein levels (Figure S4B and S4C).

To gain insight into the molecular mechanism controlling the G_2_ arrest, we checked phosphorylation of checkpoint factors (Figure 2B). ATM-Ser1981 phosphorylation (pATM) is very strong in DDB1-depleted cells likely due to the formation of DSBs, detectable by Comet assay (Figure S5), that are induced by re-replication [20], [42]. Similarly, the ATM substrate H2AX is phosphorylated on Ser139 (γH2AX), and CHK1 is strongly phosphorylated on Ser317 (pChk1). On the other hand, CHK2-Thr68 phosphorylation does not change in DDB1-depleted cells both in HeLa and U2OS cells (data not shown). Also CSN subunits depletion activates CHK1 and H2AX similarly to what observed for DDB1 depletion (Figure S6), confirming that by knocking down DDB1 we are looking at the effects of the CSN- CRL4^CDT2^ axis inactivation.

Parallel with the loss of the >4C cell sub-population (Figure 2C, Bar DDB1 CDT1), inhibiting CDT1 accumulation in DDB1-depleted cells prevents ATM activation and γH2AX formation; interestingly, CHK1 phosphorylation is still evident in these cells (Figure 2B, column DDB1 CDT1 and Figure S7B). Remarkably, notwithstanding an inactive ATM-dependent checkpoint, the G_2_ arrest is not affected by the knock-down of CDT1 in both DDB1-depleted HeLa or U2OS cells (Figure 2B and Figure S7A). Altogether, these data suggest the existence of a CDT1-independent (origin licensing-independent) function of CRL4^CDT2^, whose inactivation causes a G_2_ cell cycle arrest through an ATM-independent mechanism, which may rely on CHK1.

Treatment of DDB1- CDT1-codepleted cells with caffeine [44] prevents CHK1 phosphorylation and the accumulation of G_2_-arrested cells (Figure 2B and 2C; Figure S4A), suggesting the involvement of ATR. Indeed, ATR knockdown in DDB1-depleted cells, opposite to CDT1 knockdown in DDB1-depleted (Figure 2C, column DDB1 CDT1), reduces the 4C DNA content sub-population without affecting the >4C sub-population (Figure 2D).

Immunostaining of exponentially growing DDB1-depleted cells with anti-RPA and anti-pATM antibodies reveals two types of cell populations by immunofluorescence. Indeed, some cells show exclusively RPA foci, while other cells show RPA foci co-localizing with pATM. The representative field reported in Figure S8A shows DDB1-depleted cells where one cell (white arrow) exhibits several pATM foci many of which co-localize with RPA, and one cell (yellow arrow) with several RPA-positive but no pATM foci. It should be noticed that the morphology of the RPA foci is different in the two cell types, since those co-localizing with pATM have generally a larger size. The fraction of cells only positive for RPA foci, and that positive for both pATM and RPA are quantified in Figure S8B. These data suggest that cells where depletion of DDB1 affects origin licensing accumulate DNA damage and are characterized by pATM and RPA positive foci. On the other hand, in cells where DDB1 depletion only causes an origin licensing-independent effect, ATM is not activated and only RPA foci are detectable. Together with the data presented above, these observations suggest that the origin licensing -independent function of DDB1 may impinge on replication fork progression. Indeed, replication forks stalling leads to uncoupling of helicase and DNA polymerase activities and accumulation of single-stranded DNA (ssDNA) coated with RPA, which triggers an ATR-dependent response [45], [46].

### CRL4^CDT2^ is required for progression through S-phase

**Figure 3:**
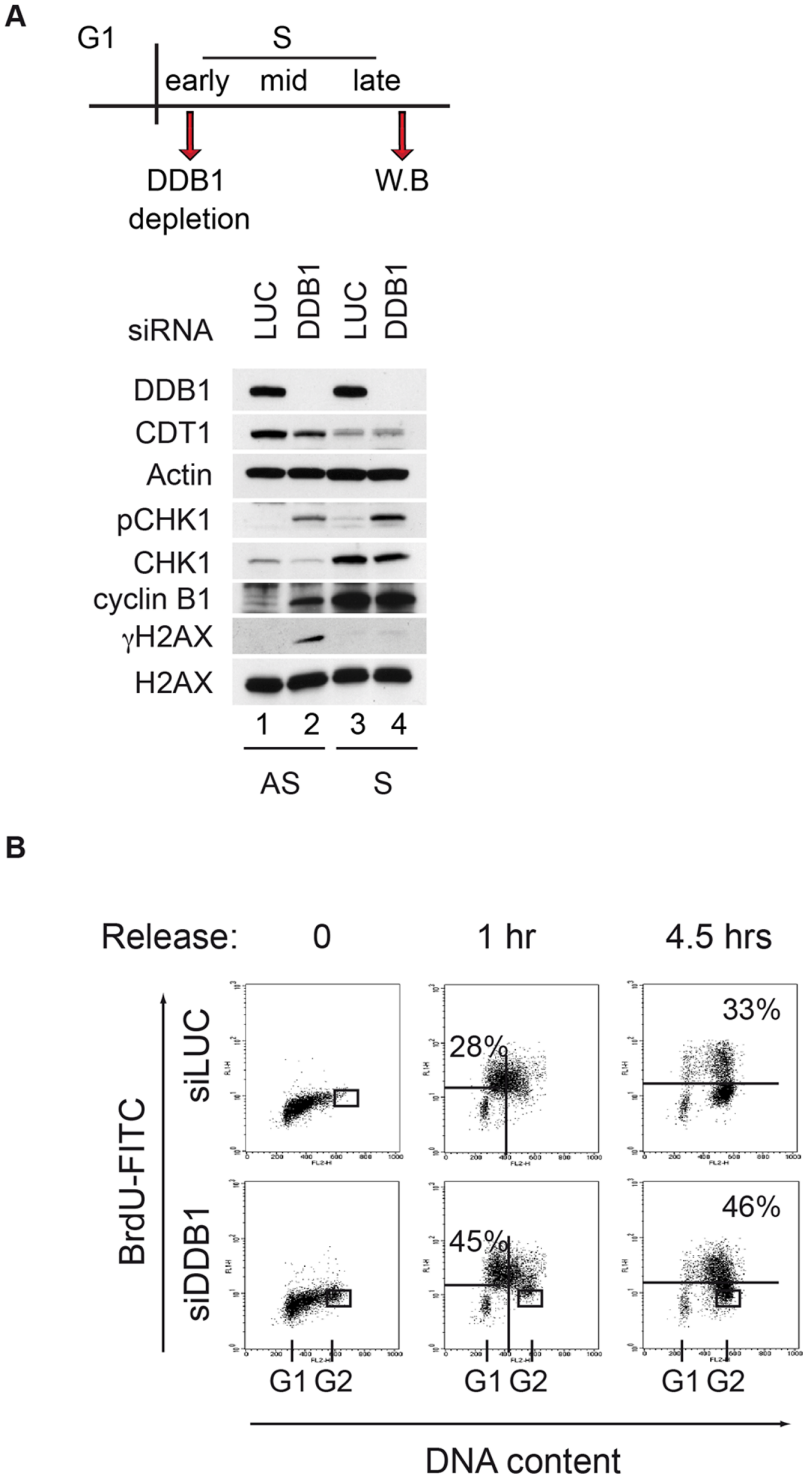
DDB1 depletion causes replication stress. (A) Total protein extracts of HeLa cells depleted with the indicated siRNAs were fractionated by SDS-PAGE and immunoblotted with the indicated antibody. AS (asynchronous) indicates exponentially growing cell total protein extracts. S (synchronous) indicates total protein extracts from HeLa cells harvested 5 hours after releasing from a DTB. The experimental set up is summarized above the immunoblot. (B) HeLa cells were transfected with control or siDDB1 during DTB. Cells were labeled with BrdU and harvested at the indicated time points following DTB. Cells were immunostained with anti-BrdU antibody and DNA content was monitored by flow cytometry using propidium iodide staining. A representative FACS profile of three independent experiments with similar results is shown. Percentage of cells incorporating BrdU in early S-phase (1 hr) or late S-phase (4.5 hrs) was calculated. A minor population of permanently arrested G2 cells was detected in DDB1-depleted cells and was not considered in quantifications (delimitated by rectangle). G_1_ indicates cells with DNA content 2C; G_2_ indicates cells with DNA content 4C.

As shown above, depletion of DDB1 in cycling cells causes re-replication, which activates an ATM-dependent signaling, but disrupts also an independent process leading to activation of ATR (Figure 2C and Figure 3A “AS”). Another approach to investigate this CDT1-independent pathway and to confirm that CRL4^CDT2^ may play a role in S-phase also after origin firing, is to set up a DDB1 depletion protocol that allows CRL4^CDT2^ downregulation after it has promoted CDT1 removal from chromatin, so that re-replication is not induced. HeLa cells were synchronized in early S-phase by a double thymidine block (DTB) [47]; downregulation of DDB1 was achieved by treating cells with siRNA five hours before the second thymidine addition, so that DDB1 down-regulation occurs when the majority of the cells are indeed in early S-phase and CDT1 at origins has already been degraded by CRL4^CDT2^ (Figure 3A, scheme at the top). Using this experimental strategy, Figure 3A confirms that 5 hrs after the release from DTB, when cells have completed S-phase and entered G_2_ as shown by the elevated levels of cyclin B1, CDT1 levels are low and DDB1 depletion does not cause an increase in CDT1 compared to the control (compare “AS” and “S”). These results are consistent with previous work suggesting that during S-phase and G_2_-phase, CDT1 levels are also controlled by CUL1 [31], [32].

Figure 3A also shows that 5 hrs after DTB release, siLUC-treated control cells were in G_2_, as indicated by the elevated levels of cyclin B1, while CHK1 and H2AX were not phosphorylated (Figure 3A, “S”). On the other hand, DDB1-depleted cells, while showing similar G_2_-phase markers, activate CHK1 without phosphorylating the ATM target H2AX (Figure 3A, “S”). These results demonstrate that loss of DDB1 function leads to γH2AX formation only in asynchronous cells and not in S-phase cells, where only CHK1 is phosphorylated (lanes 2, 4; Figure 3A), confirming that disruption of the CDT1-independent function of CRL4^CDT2^ during S-phase causes activation of an ATM-independent DDR response.

To characterize this CDT1-independent role of CRL4^CDT2^ during S-phase, we monitored BrdU incorporation at different time-points following DTB release. At time 0, when cells are arrested by DTB, both control cells (siLUC) and DDB1-depleted cells (siDDB1) do not incorporate BrdU and are mainly in early S-phase (Figure 3B). 1 hr following DTB release, most of the BrdU incorporating siLUC cells were in mid S-phase with a small proportion of cells in early S-phase (28%), while DDB1-depleted cells were delayed (45% still in early S). 4.5 hrs after DTB release, the majority of control cells are in G_2_ and do not incorporate BrdU, and approximately 33% of the cells are still in late S-phase and actively replicating. At the same time point a higher percentage (46%) of DDB1-depleted cells are still incorporating BrdU, and are spread over middle and late S-phase (Figure 3B). These data indicate that DDB1 depletion affects a timely progression of replication forks during S-phase.

### CRL4A^CDT2^ and CRL4B^CDT2^ interact with each other and CSN to carry out non-redundant functions In S-phase

**Figure 4:**
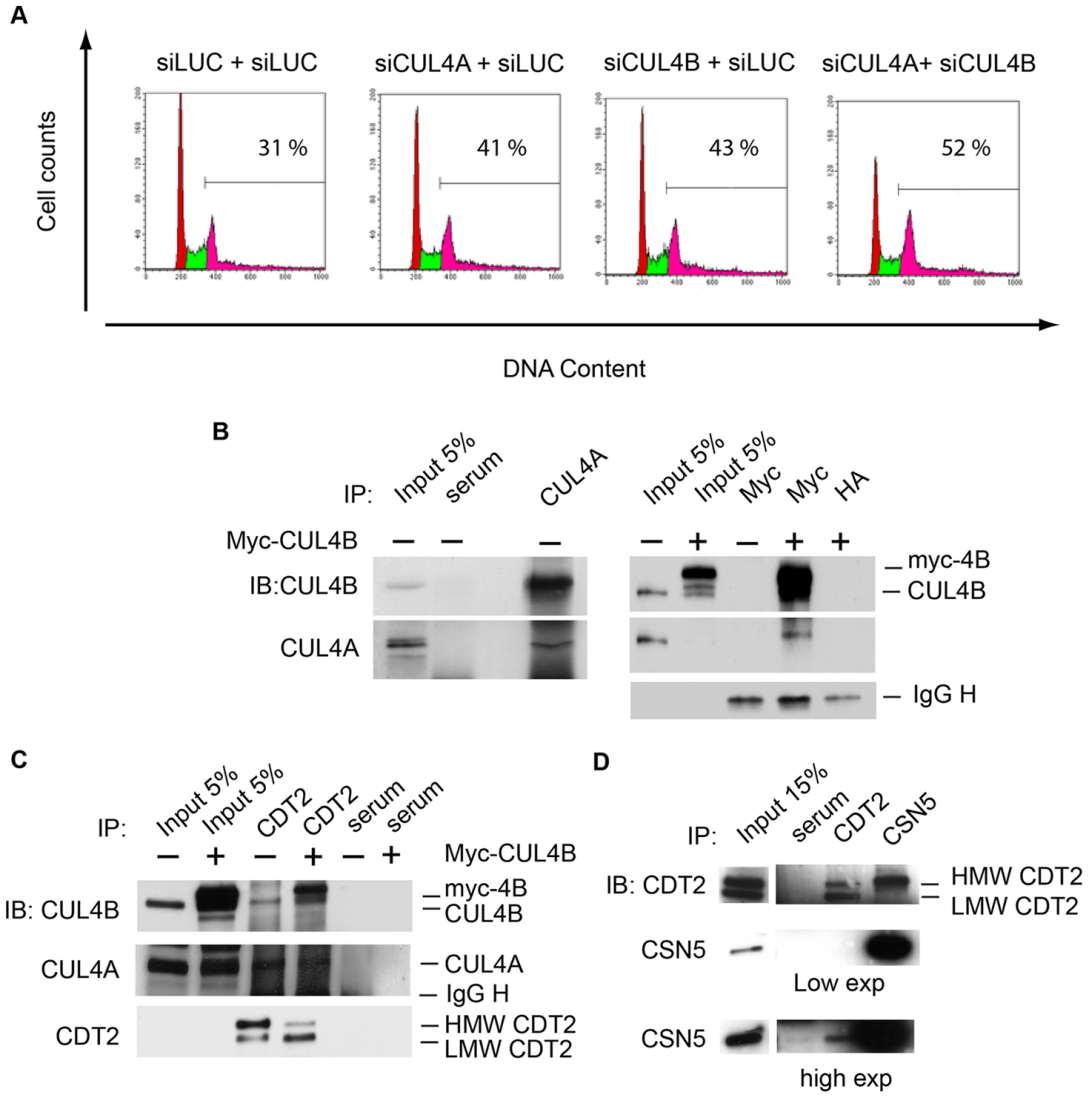
CSN-CRL4^CDT2^ contains both CUL4A and CUL4B in S-phase. (A) Cell cycle distribution of HeLa cells transfected with the indicated siRNAs was analyzed by DNA content flow cytometry detection. A representative FACS profile is shown. Percentage of cells in G_2_ with a 4C or >4C DNA content were calculate by Cell Quest software. (B) (C) (D) HeLa cells were synchronized by DTB and, where indicated, transfected with myc control or myc-CUL4B vectors in between the two thymidine blocks. Cells were harvested at 2.5 hrs after DTB release and total cell protein extracts were prepared. Cell lysate was subjected to immunoprecipitation with serum as control or with the indicated antibodies. Immunoprecipitates and cells lysates were immunoblotted with the indicated antibodies. (D) Both low and high exposure films are shown. HMW is High Molecular weight; LMW is low molecular weight.

Generally, CRL4 complexes containing CUL4A are considered to have overlapping functions with CRL4 complexes containing CUL4B. If CUL4A and CUL4B are part of independent complexes with overlapping function, depletion of either CUL4A alone or CUL4B alone, are not expected to cause any major phenotype, while concomitant depletion of both should have an effect. The observation that depletion of either CUL4A or CUL4B prevents degradation of CDT1 (Figure S4B) suggests that the two proteins do not have redundant functions in the degradation of CDT1. This is consistent with the two proteins working in the same linear pathway, or with CUL4A and CUL4B participating to the same CRL4^CDT2^ complex, which may have a non-canonical architecture. Consistently, RNA interference in U2OS cells shows that each single CUL4A and CUL4B siRNA induces a 4C and >4C G_2_ arrest of comparable penetrance. The double siRNAs show a higher penetrance, likely due to the double amount of siRNAs used (Figure 4A). These genetic data may suggest the presence of a single CRL4 complex containing both CUL4A and CUL4B (CRL4A/4B^CDT2^) whose depletion causes the phenotype described.

To support these data with biochemical evidence, HeLa cells were synchronized by DTB, transfected with a control vector or a vector expressing myc-tagged CUL4B, collected in mid S-phase and total protein lysates analyzed by coimmunoprecipitation (co-IP) assays. Anti-CUL4A antibodies immunoprecipitate endogenous CUL4A and co-IP endogenous CUL4B; anti-myc antibodies immunoprecipitate myc-tagged CUL4B and co-IP endogenous CUL4A and CUL4B (Figure 4B). An anti-CDT2 Ab, but not a control rabbit serum, immunoprecipitates CDT2 and co-IP endogenous CUL4A and CUL4B, and exogenous myc-CUL4B (Figure 4C). The resistance of such interactions to ethidium bromide excludes they may be mediated by DNA (Figure 4 and not shown). Thus, genetic and biochemical data suggest collectively the possible existence of a complex containing CUL4A, CUL4B and CDT2.

A subset of CRLs are assembled to, and regulated by, CSN [48]. We have observed that CSN depletion induces the same phenotype observed for CRL4^CDT2^ subunits depletion, suggesting that CRL4^CDT2^ may be physically complexed to CSN. Indeed, a CDT2 antibody (Ab) co-IP CSN5 and a CSN5 Ab selectively co-IP HMW-CDT2 (Figure 4D), suggesting a more complex organization of CRL4^CDT2^.

### PRR and CRL4A/4B^CDT2^ cooperate in preventing apoptosis due to incomplete DNA replication

**Figure 5:**
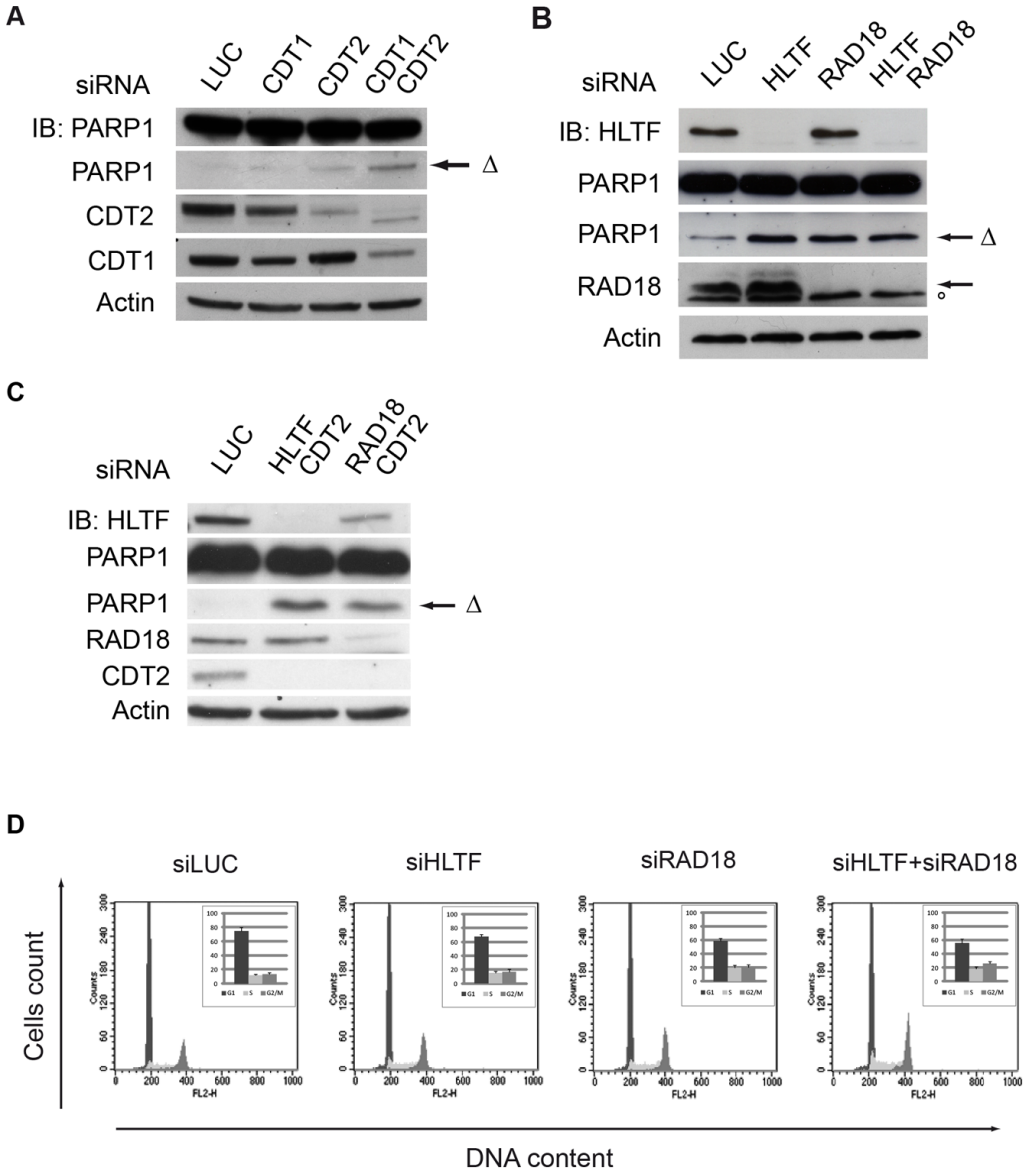
CRL4A/4BCDT2 is functionally linked to RAD18 and HLTF in S-phase. (A, B, C) HeLa cells were subjected to three rounds of transfection with the indicated siRNAs. Both detached and adherent cells were harvested 48 hours after the last round. Total protein lysates were analyzed by immunoblotting with the indicated antibodies. Δ indicates caspase3-cleaved PARP1 fragment. (°) indicates a background band. (D) Cell cycle distribution of HeLa cells transfected with the indicated siRNAs was determined by DNA content flow cytometry detection. Number of cells in each cell cycle phase was quantified. Relative values were represented as bars. Each bar represent the average of three independent experiments and the error bars represent the SDs.

We noticed that loss of the CDT1-independent function of CRL4A/4B^CDT2^ in exponentially growing cells induces an increase in detached cells over mock, a possible indication of apoptosis (not shown). Indeed, 48 hrs after siRNA transfection, CDT2-depletion or concurrent CDT2- and CDT1-depletion induce apoptosis, as detected using an Ab recognizing both full length and caspase3-cleaved PARP1 (ΔPARP1) (Figure 5A and Figure S9A). The ΔPARP1 signal appears stronger when CDT2 and CDT1 are simultaneously depleted, this may suggest that the apoptotic phenotype is induced prevalently by inactivation of the CDT1-independent function of CRL4A/4B^CDT2^, or that CDT1 and CDT2 have a synthetic effect. Previous work suggested that CRL4^CDT2^ is required for error-prone bypass of UV-induced DNA lesions [41]. We hypothesized that the CDT1-independent function of CRL4^CDT2^ described above and required for proper S-phase progression may be linked to its role in PRR during normal DNA replication. To test this hypothesis we checked whether depletion of RAD18 or HLTF would also lead to an apoptotic phenotype. Depletion of RAD18, HLTF or both in HeLa or U2OS cells generates apoptotic ΔPARP1, and an increase in cell mortality over mock depleted cells estimated ∼70%, ∼25% and ∼50%, respectively (Figure 5B, Figure S9B and S9C).

To determine the relationship between the anti-apoptotic roles of CRL4^CDT2^ and of RAD18/HLTF, we analyzed apoptotic markers in cells concomitantly depleted of either CDT2 and RAD18 or CDT2 and HLTF. Depletion of CDT2 in either RAD18- or HLTF-depleted cells does not exacerbate their apoptotic phenotype suggesting that CDT2 and RAD18 may operate together in preventing apoptosis (Figure 5C).

The apoptotic phenotype of cells depleted for PRR factors likely derive from problems arising during a normal S-phase. This was confirmed by the observation that cells depleted for RAD18, HLTF or both showed a G_2_ accumulation supporting the notion of a failure in proper completion of DNA replication (Figure 5D).

### CRL4^CDT2^ interacts with RAD18 during normal S-phase progression and modulates its recruitment to chromatin

After UV-induced DNA damage, CRL4^CDT2^ was shown to modulate PRR controlling a RAD18-independent PCNA monoubiquitylation [41]. We investigated the mechanism through which CRL4^CDT2^ cooperates with PRR factors during a normal S-phase.

**Figure 6:**
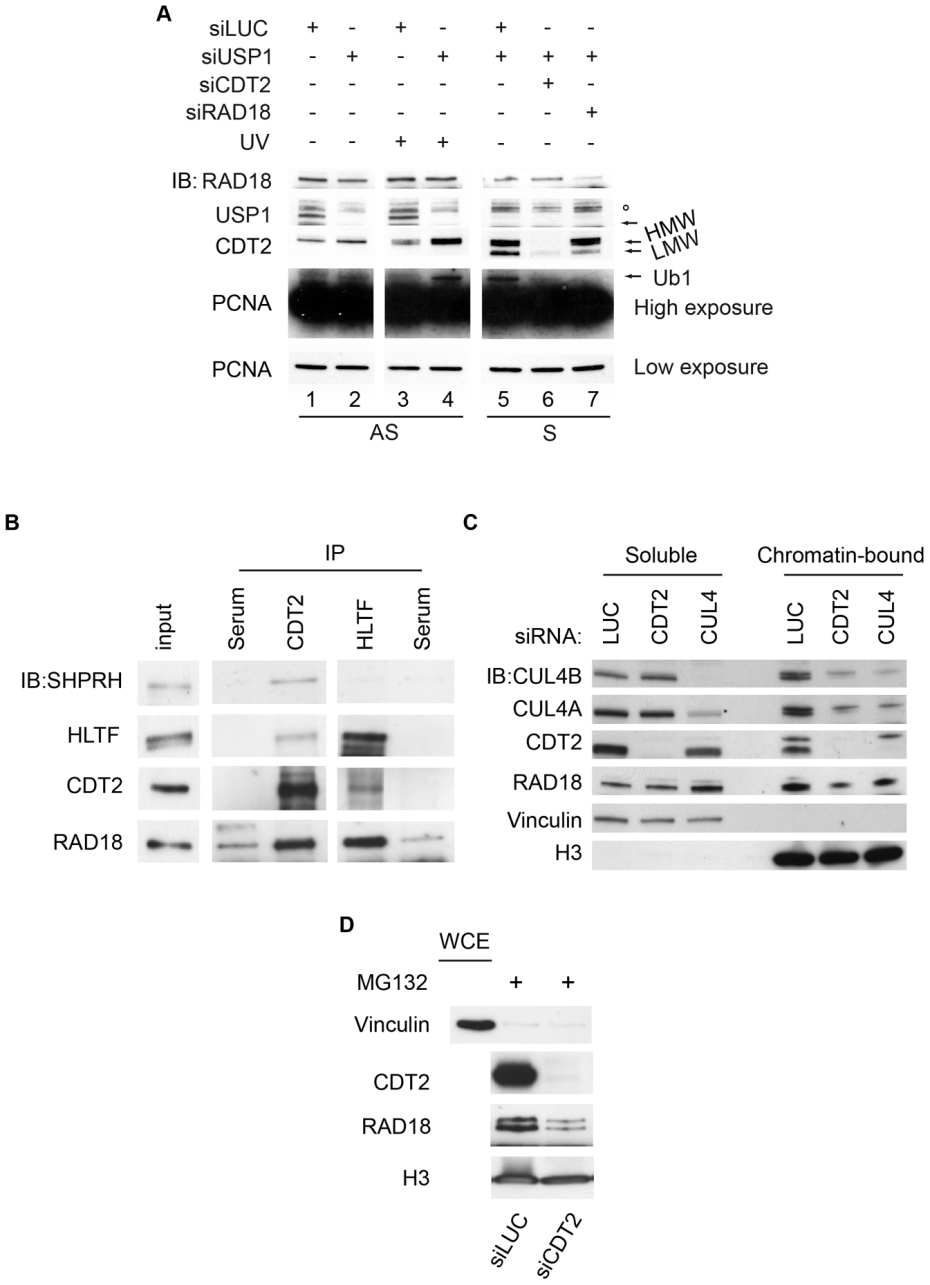
CRL4A/4B^CDT2^ modulates RAD18 binding to chromatin and PCNA monoubiquitylation. (A) HeLa cell total protein lysates were resolved by SDS-PAGE and immunoblotted with the indicated antibodies; (AS) exponentially growing HeLa cells transfected with the indicate siRNAs, mock or UV-irradiated; (S) HeLa cells were synchronized in mid-S-phase after transfection with the indicated siRNAs as in Figure 3A. HMW and LMW indicates respectively slow and high migrating CDT2; Ub1 indicates mono-ubiquitin-PCNA; ° indicates a not specific band. (B) HeLa cells were synchronized in mid-S-phase, protein-protein cross-linked and harvested. Total cell protein extract was prepared. Cell lysate was subjected to immunoprecipitation with serum as control or with the indicated antibodies. Total cells lysate (input,10%) and immunoprecipitates were immunoblotted with the indicated antibodies (C) (D) HeLa cells were transfected with the indicated siRNAs as in Figure 3A, synchronized and harvested in mid-S-phase. Total protein extract were fractionated into soluble and chromatin-bound fractions, resolved by SDS-PAGE and immunoblotted with the indicated antibodies (D) MG132 was added for two hours prior to harvesting where indicated.

In exponentially growing (AS) HeLa cells, ubiquitylated PCNA is barely detectable provided that the deubiquitylating enzyme USP1 [49] is downregulated (Figure 6A, lanes 1–2), while ubiquitylation is evident after induction of exogenous DNA damage by UV exposure (Figure 6A, lanes 3–4). Differently from what observed in logarithmically growing cells, PCNA ubiquitylation in USP1-depleted cells is clearly detectable during an unperturbed S-phase (S). In these conditions, PCNA is monoubiquitylated in mid S-phase following DTB release and this modification is greatly reduced by depletion of CDT2 (Figure 6A, compare lanes 5 and 6). RAD18 depletion prevents PCNA monoubiquitylation (Figure 6A, lane 7), as previously reported [50]. We also noted that two forms of CDT2 (HMW- and LMW) are detectable in mid S-phase cells, and treatment with a siRNA against the CDT2 coding sequence depletes cells of both HMW- and LMW-CDT2, confirming that they are indeed CDT2 isoforms (Figure 6A, panel S, lines 5, 6, and Figure 4C).

These results may suggest that either CRL4A/4B^CDT2^ modulates RAD18-dependent PCNA ubiquitylation, in agreement with our genetic data indicating that CRL4A/4B^CDT2^ and RAD18/HLTF function together during S-phase, or that RAD18 and CRL4A/4B^CDT2^ additively control PCNA ubiquitylation.

In order to assess whether CDT2 is physically associated to the RAD18/HLTF and the RAD18/SHPRH complexes, protein-protein interactions were analyzed by coimmunoprecipitation experiments using total protein extracts from HeLa cells that were synchronized in mid S-phase [39]. Immunoprecipitation of CDT2 co-immunoprecipitates RAD18 and both HLTF and SHPRH; immunoprecipitation of HLTF co-immunoprecipitates RAD18 and CDT2 but, as expected, not SHPRH (Figure 6B). Indeed, it has reported that binding of HLTF and SHPRH to RAD18 are mutually exclusive [39]. These results indicate that CDT2 binds to both the RAD18/HLTF complex and the RAD18/SHPRH complex in S-phase. Given that we did not observe an increase of the steady-state levels of RAD18 when we knocked down CDT2 (Figure 6A and 6C), it is unlikely that CRL4A/4B^CDT2^ marks RAD18 for degradation. Remarkably, downregulation of CRL4A/4B^CDT2^ in HeLa cells synchronized in mid S-phase led to a reduction in the amount of chromatin-bound RAD18 (Figure 6C), which appears to be independent of the degradative activity of the proteasome, since addition of the MG132 proteasome inhibitor does not affect the decrease in chromatin-bound RAD18 after CDT2 depletion (Figure 6D). Interestingly, we noticed that RAD18 depletion shifts the ratio between HMW- and LMW- CDT2 compared to the control (Figure 6A, compare line 6 to line 7), indicating a further layer of complexity in the relationship between CDT2 and RAD18. Altogether, our findings suggest that CRL4A/4B^CDT2^ may facilitate binding of RAD18-containing complexes to chromatin.

## Discussion

In this paper we describe a new regulatory mechanism that modulates TLS DNA synthesis during a normal S-phase, possibly as a consequence of spontaneous DNA damage sensed by replication forks. A large complex consisting of CRL4^CDT2^ and containing both CUL4A and CUL4B regulates the recruitment of RAD18 to chromatin and controls PCNA monoubiquitylation.

### CRL4^CDT2^ is important for proper S-phase completion in an unperturbed cell cycle

CRL4^CDT2^ promotes degradation of factors involved in replication origins licensing, particularly CDT1, once the origin has been fired. This activity is important to prevent reassembly of a potential pre-replication complex; indeed inactivation of CRL4^CDT2^ leads to re-replication within the same cell cycle (for a review see [21]). A second role for CRL4^CDT2^, independent form CDT1, was hypothesized but never investigated [42].We show that after origins have been fired, and after origin–bound CDT1 degradation has been completed, inactivation of CRL4^CDT2^ does not result in further changes in the residual CDT1 levels, but affects DNA replication by slowing down S phase progression and by triggering a checkpoint, likely dependent on ATR. These observations suggest that during DNA replication, after origin firing, CRL4^CDT2^ may have other targets, exerting a CDT1-independent function.

**Figure 7:**
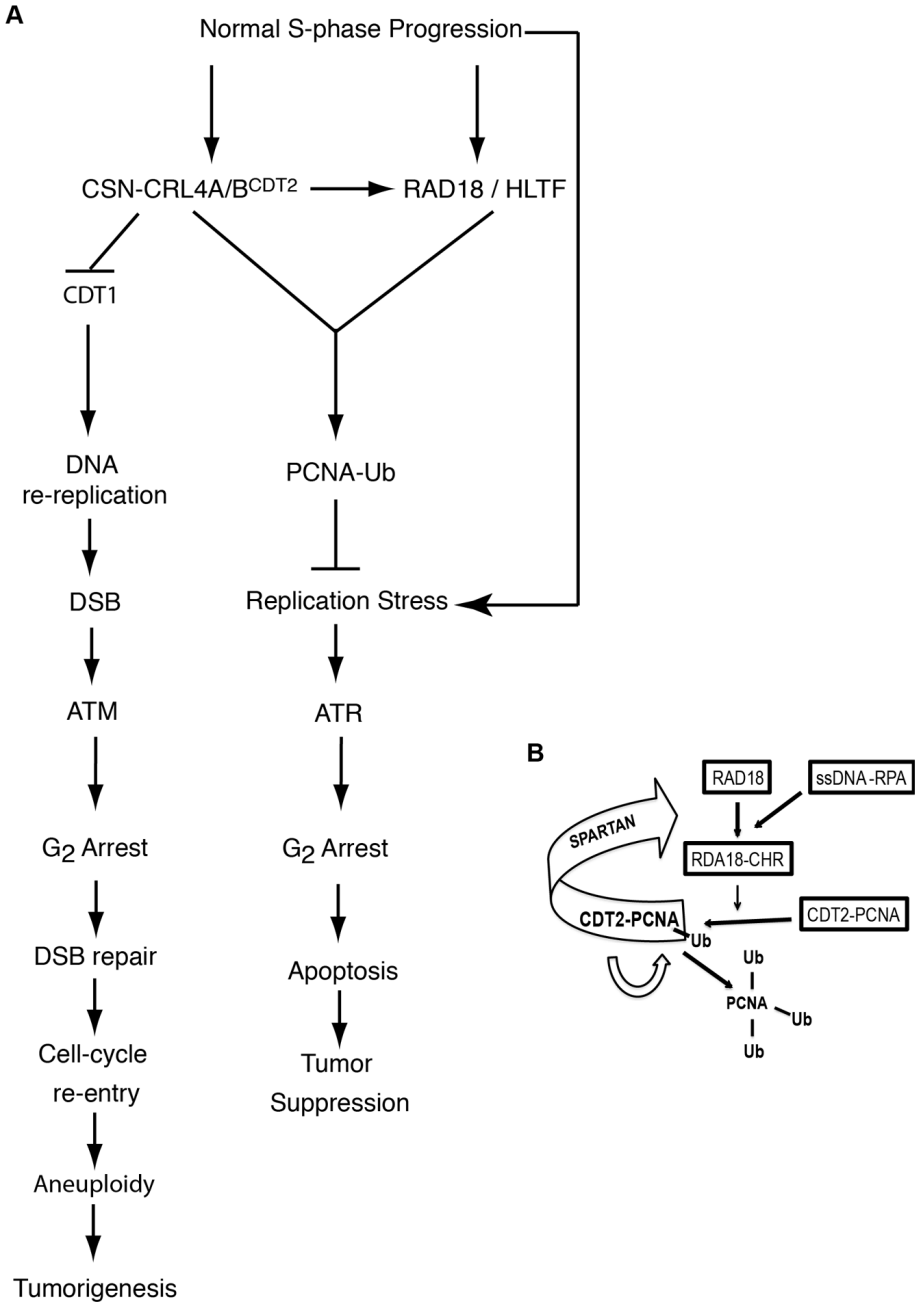
CSN-CRL4A/4B^CDT2^ functions in S-phase. CSN-CRL4A/4B^CDT2^ is required in two different steps of the DNA replication during a normal cell cycle. (A) At the G_1_/S transition CSN-CRL4A/4B^CDT2^ is required to prevent DNA origins re-firing by means of a variety of molecular mechanisms. Failure to do so causes DNA re-replication, DSBs and ATM signaling activation. Eventually, DSBs are repaired and cells with re-replicated DNA re-enter the cell cycle and divide. Mitosis of cells with re-replicated DNA causes chromosomal aberrations that may drive tumorigenesis. Following origins firing, replication-linked DNA damage activates regulative loops between the ATR- dependent checkpoint and PRR. Inactivation of this pathway causes permanent replication stress, ATR hyper-signaling and eventually apoptosis. The relevance of this pathway makes it a pharmacological target to potentially induce replication stress and apoptosis in cancer cells. (B) CSN-CRL4A/4B^CDT2^ regulates RAD18 recruitment to chromatin, possible in cooperation with Spartan, in a feed-forward loop leading to the amplification of PCNA ubiquitylation and consequent error-free and error-prone TLS. CSN-CRL4A/4B^CDT2^ may also contributes to the amplification of PCNA ubiquitylation by directly mono-ubiquitylating PCNA.

Inactivation of CRL4^CDT2^ in exponentially growing cells has been previously shown to leads to a DDR activation phenotype [22], [42]. Our data suggest that this is likely due to the combination of two independent checkpoint pathways. Indeed, DNA re-replication caused by failure to degrade CDT1 after CRL4^CDT2^ inactivation causes DSBs and triggers an ATM- CHK1- dependent G_2_ checkpoint, which is p53 independent. On the other hand, impairment of the CDT1-independent CRL4^CDT2^ function leads to replication stress and to a G_2_ arrest, which is ATR- CHK1- dependent and distinct from the pathway induced by DNA re-replication (Figure 7A)

Accordingly with the idea that DNA re-replication is a cancer inducer [29], we also show that cells with overreplicated DNA induced by CRL4A/4B^CDT2^ inactivation escape apoptosis.

### The CRL4^CDT2^ complex contains both CUL4A and CUL4B

Having outlined a new relevant role of CRL4^CDT2^ during normal S-phase progression, we investigated the involvement of CUL4A and CUL4B in this activity. We found that in S-phase CUL4A and CUL4B act, genetically, within the same pathway. This observation expands the current view that two physically independent CUL4 ubiquitin ligases complexes, one with CUL4A and the other with CUL4B, exist and have overlapping substrates and functions. The genetic data, together with a detailed co-IP analysis, suggest that a complex containing both CUL4A and CUL4B, which we indicate as CRL4A/4B^CDT2^, carries out two functions in S-phase: preventing DNA re-replication and tolerating spontaneous DNA damage. There are two potential mechanisms for CUL4A-CUL4B dimerization: substrate receptor subunit-mediated dimerization and Nedd8-cullin linkage [51], [52], [53]. We do not know whether CUL4A and CUL4B directly dimerize in CRL4A/4B^CDT2^ or whether a CRL4A^CDT2^ complex binds to a CRL4B^CDT2^ complex through CDT2 dimerization. Further biochemical studies will be required to address this question. Nevertheless, complex formation and subunit dimerization may represent a new regulatory mechanism of CRL4A/4B ^CDT2^ activity during replication.

### CSN regulates the CRL4A/4B ^CDT2^ activity during DNA replication

Our data provide insights on the role of the CSN complex in regulating CRL4A/4B ^CDT2^ activities during S-phase, helping to clarify controversial reports on its involvement in modulating CDT1 stability after DNA damage [7], [10]. We show that CSN is physically associated to CRL4A/4B^CDT2^ in S-phase. Moreover, CSN regulates origin licensing, targeting CDT1 for degradation at the G_1_/S transition; indeed loss of CSN function induces DDR activation, G_2_ arrest and promotes re-replication similarly to what observed by depleting CRL4A/4B^CDT2^ subunits.

### CRL4^CDT2^ regulates binding of RAD18 ubiquitin ligase to chromatin and modulates PCNA ubiquitylation

Previous reports indicated that CRL4^CDT2^ has an important role in promoting PRR during replication of a UV damaged template [41], where CRL4^CDT2^ is responsible for PCNA monoubiquitylation in a RAD18-independent pathway [41]. We hypothesized that the CDT1-independent function of CRL4^CDT2^, may be involved in modulating PRR also during a normal S-phase, in the absence of exogenously induced UV damage. Indeed, we show that during S-phase CRL4^CDT2^ physically interacts with PRR factors (i.e. RAD18, HLTF, SHPRH). In our experimental conditions, CDT2 depletion greatly reduces the RAD18-dependent S-phase specific monoubiquitylation of PCNA. The hypothesis that CRL4^CDT2^ may control PRR through its interaction with RAD18 is further supported by the observation that, while CDT2 depletion does not affect the steady-state level of RAD18, as previously reported [41], it decreases the levels of chromatin-bound RAD18 in S-phase, likely through the capacity of chromatin-bound CDT2 to interact with RAD18/HLTF and the RAD18/SHPRH complexes.

CRL4^CDT2^ activity is coupled to DNA synthesis through its binding to PCNA. PCNA-bound CRL4^CDT2^ oversees the degradation of licensing factors at replication origins [3], [25], [26], [42], acts in the resolution of replication forks stalled at topo I-DNA complexes [9] and, in the *C. elegans* embryo, controls the removal of pol η from replication forks after TLS [43]. These data suggest a model in which CRL4^CDT2^ moves along with PCNA during replication fork progression to act as a molecular machine committed to resolve sources of replication stress. The existence of a feed-forward loop that amplifies the response leading to PCNA ubiquitylation has been described very recently [40]. A key player in this regulatory circuit is Spartan, which binds monoubiquitylated PCNA and, by modulating RAD18 function, enhances PCNA ubiquitylation and promotes TLS. Our findings, showing a very similar role for CRL4A/4B^CDT2^, integrate this model and suggest that CRL4A/4B^CDT2^, through protein-protein interactions, enhances the binding of RAD18 to selected sites where replication may be problematic, and amplifies the formation of monoubiquitylated PCNA to facilitate replication via TLS (Figure 7B).

Inactivation of the CSN-CRL4A/4B^CDT2^, RAD18, HLTF pathway reduces cell survival and promote apoptosis, suggesting that PRR may be considered as a potential target for cancer therapy.

## Materials and Methods

### Cell Culture

HeLa and U2OS (ATCC HTB-96) cell lines were cultured in DMEM containing 10% FBS, penicillin, streptomycin and L-Glutammine and kept at 37°C in a humidified atmosphere with 5% CO_2_.

### Antibodies

Antibodies anti-pChk1 (Ser317), anti-RPA70 and anti-53BP1 were from Cell Signaling Technology, anti-Chk1 and anti-Actin, anti-DDB1, anti-CSN5, anti-CUL4B from Sigma, anti-CSN2 and PCNA from Calbiochem and anti-BrdU was obtained from BD Biosciences; anti-ATM, anti-pCdc2 (Tyr14), anti-CDT2, anti-H3, anti-pH3 (Ser10) and ATR, cyclin B1 (CycB1) from AbCam; anti-γH2AX clone JBW301, anti-H2AX, anti-pATM (Ser1981) were purchased from Upstate, anti-PARP (H-250), anti-CDT1(H-300), anti-cyclin A (H-432) were from Santa Cruz Biotechnology, anti-CUL4A was from Rockland; USP1 was a gift from Fanconi Anemia Foundation.

### UV and drugs treatment

Caffeine (Sigma) was used at 5 mM for 24 hrs. For UV irradiation, medium was removed, cells were washed once with PBS, and then irradiated with UV Stratalinker (predominantly 254 nm) at a final dose of 50 J/m^2^. Subsequently, the medium was added back to the cells and the cells returned to culture conditions for 1 h. To inhibit the proteasome activity, MG-132 (Sigma) was added at 100 µM two hours prior to harvesting.

### Protein depletion and Double Thymidine Block (DTB)

For exponentially growing, cells were seeded at low density in plates and subjected to serial cycles of siRNA transfection; 48 h after the last transfection, cells were harvested for western blotting analysis, immunofluorescence and FACS analysis. For G_1_/S-phase synchronization, HeLa cells were subjected to Double Thymidine Block (DTB) as following: thymidine (Sigma), at a final concentration of 2 mM, was added to a low density plated cell culture for 19 hrs. Cells were then washed 3 times with DMEM followed by a 9 hrs release. 2 mM Thymidine was added back for 16 hrs; 5 hrs before the second thymidine addition, liposome complexed siRNAs were added. Cells were then released for the indicated period of time. siRNA were purchased from MWG and Lipofectamine 2000 Transfection Reagent from Invitrogen.

### FACS analysis

Cells were harvested and washed in PBS, fixed in 70% ice cold EtOH and either stained with propidium iodide (PI) at room temperature or processed for anti-BrdU or for anti-pH3 (Ser10) (with AlexaFluor 488 as secondary antibody) immunolabeling to determine S-phase, M phase and re-replicating cells. FACS analysis were performed on a BD FACScan and quantified with Cell Quest software (BD Bioscience). 10^4^ events were acquired and the same number is visualized in the PI histograms while 50% of the total events are shown in the BrdU/PI Dot Plot.

### Immunoprecipitation

HeLa cells were synchronized in mid S-phase by DTB as described. Total cell lysates were prepared by solubilizing cells in LYSIS BF (420 mM NaCl, 50 mM Tris-HCl pH 7.5, 1% NP-40.5 mM MgCl_2_, phosphatase and protease inhibitors). After extraction, Lysates were diluted to 150 mM NaCl. 0.5–0.8 mg of protein extract were used for each immunoprecipitation. To co-immunoprecipitate CDT2 with PRR factors from chromatin, the protocol described in [39] , which requires protein-protein cross-link, was employed. Immunoprecipitates were analysed by 4–20% SDS-PAGE.

### Whole cell extraction and subcellular fractionation analysis

For total protein extracts analysis, cells were lysed in 1% SDS sample buffer (62.5 mM Tris-HCl, pH 6.8, 2% wt/vol SDS, 10% glycerol, 50 mM DTT, 0.01% wt/vol bromophenol blue), heated at 95°C for 5 min, sonicated 10 sec, and high speed supernatant was analysed by 4–20% SDS-PAGE. For Chromatin fraction analysis cells were processed as previously reported in [54]

### Immunofluorescence

Cells depleted of the indicated proteins, were seeded on a coverslip. At the indicated time point, cells on coverslips were washed once in PBS, fixed 20 min with 2% paraformaldhyde (PFA) in PBS and permeabilized with ice cold PBS containing 0.2% Triton X-100 for 5 min (for detection of RPA foci positive cells permeabilization was performed before fixation). Blocking was performed in 10% BSA in PBS for 30 min and subsequently replaced with primary antibody diluted in PBS with 0.1% TWEEN 20 (PBST 0.1) for 2 h at room temperature. Coverslips were washed three times in PBST 0.1 for 10 min and secondary antibody diluted in PBST 0.1 was added (anti-mouse AlexaFluor 488 and anti-rabbit AlexaFluor 594); nuclei were counterstained with DAPI. Cells were rinsed in PBST 0.1 three times for 10 min and mounted using ProLong Gold (Invitrogen).

Images were taken using a Leica DMRA2 Microscope with a 100× objective and a ViCo microscope with a 60× or 100× magnification objective (Nikon).

53BP1 foci positive cells were counted randomly over the whole coverslip and at least 150 cells were scored for each treatment. The number of foci per cell was scored using ImageJ software (threshold = 35, pixel∧2 = 5-infinity, circularity = 0.00–1.00) and at least 50 cells were analyzed for each siRNA transfected sample.

### Comet assay

The alkaline comet assay was performed according to the Trevigen Kit Manual; briefly, after 30′ in alkaline solution, The electrophoresis was carried out in alkaline solution at 1 V/cm for 30 min at 4°C. Images were obtained using a Zeiss Axioskop and subsequently analyzed with Comet Score Software (TriTeck Corporation), giving the different parameters of the images.

### Cytotoxicity assay and apoptosis

Cells were seeded in 96 well plate and analyzed at the indicated time points following the last siRNA transfection cycle. CytoTox 96® Non-Radioactive Citotoxicity Assay (Promega) was used to quantify the ratio of live and dead cells by measuring LDH into attached cells (live cells) over LDH release into medium culture (dead cells). The procedure was performed following manufacturer instructions.

Protein extracts used to evaluate the contribution of apoptosis by PARP1 and on cell mortality were prepared as follows. Detached cells in the culture media and trypsinized attached cells were pooled and harvested. The pellet was then lysed in 1% SDS sample buffer (62.5 mM Tris-HCl, pH 6.8, 2% wt/vol SDS, 10% glycerol, 50 mM DTT, 0.01% wt/vol bromophenol blue), sonicated 10 sec, and heated to 95°C for 5 min.

## Supporting Information

Figure S1 **CSN-CRL4s depletion induces 53BP1 foci with different penetrance and severity and an increase in γH2AX.** (A) HeLa cells were treated and analyzed by IF as in Figure 1A. For each independent experiment cells were scored as positive for 53BP1 foci if containing >5 foci. At least 50 cells per independent experiment were scored. Number of 53BP1 foci per cell were also counted. In the left panel it is graphically displayed the percentage of 53BP1 foci positive cells. In the right panel the mean of 53BP1 foci number per cell is graphically displayed as bar. Data represent mean +S.D. of three independent experiments. (B) Aliquots of the same HeLa cell populations in Figure 1A were harvested and cell lysates were resolved by SDS-PAGE and immunoblotted with the indicated antibodies.
(TIF)

Figure S2 **CSN-CRL4^CDT2^ depletion induces cell cycle block in G_2_ phase and re-replication.** (A) HeLa cells were depleted of the indicated proteins and cell cycle distribution was analyzed by DNA content flow cytometry detection following propidium iodide staining. A representative FACS profile of many independent experiments with similar results is shown. (B) HeLa cells transfected with the indicate siRNAs were fixed and stained with anti–Ser10-phospho-H3 primary antibody, Alexa 488-conjugated secondary antibody, and PI. Cells were analyzed by FACS. The mitotic cells in square are positive for phospho-H3. (C) HeLa cells synchronized by DTB in mid S-phase and analyzed for IF in Figure 1C, were checked for cell cycle phase by FACS analysis and for protein depletion by immunoblotting.
(TIF)

Figure S3 **Either DDB1- or CDT2-depleted U2OS cells show DDR activation and cell cycle delay.** U2OS cells depleted of the indicated proteins were harvested for further analysis. (A) Cells were fixed and stained with antibodies to H2AX phospho-S139 (γH2AX) and 53BP1; the nucleus was counterstained with DAPI. A fluorescent image of a representative nucleus is shown. A Cell sample was employed to check protein depletion by immunoblotting. (B) The cell cycle distribution was analyzed by DNA content flow cytometry detection following propidium iodide staining. A representative FACS profile with percentage of cells in each cell cycle phase of three independent experiments with similar results is shown.
(TIF)

Figure S4 **CSN-CRL4^CDT2^ has CDT1-dependent and CDT1-independent functions.** (A) HeLa cells were transfected with the indicated siRNAs (± caffeine). Cell cycle distribution was analyzed by BrdU incorporation and DNA content flow cytometry detection. (B) 48 hrs following the last transfection cycle with control (siLUC), CUL4A (siCUL4A), CUL4B (siCUL4B) or both CUL4A and CUL4B (siCUL4) siRNAs, HeLa cells were harvested and processed for SDS-PAGE. Immunoblotting was performed with the indicated antibodies. (C) 48 hrs following the last transfection cycle with control (siLUC) and CSN5 (siCSN5) siRNAs, HeLa cells were harvested and processed for SDS-PAGE. Immunoblotting was performed with the indicated antibodies.
(TIF)

Figure S5 **DDB1-depleted cells show DSBs.** Alkaline comet assay on control and DDB1-depleted HeLa cells. A graphical representation of the mean percentage of cells with tail moment >3 as DNA damage parameter is shown. Mean value and error were calculated on three independent experiments.
(TIF)

Figure S6 **CSN depletion activates checkpoints**. HeLa cells were harvested 48 hrs after the last transfection cycle with control (siLUC) or both CSN2 and CSN5 siRNA (siCSN). Total protein lysates were fractionated by SDS-PAGE and immunoblotted with the indicated antibody.
(TIF)

Figure S7 **DDB1-depleted U2OS cells show a CDT1-dependent and a CDT1-independent delay in G_2_**. U2OS cells were harvested 48 hrs after the last transfection cycle with control (siLUC), DDB1 (siDDB1) or both DDB1 and CDT1 (siDDB1+siCDT1) siRNAs and subjected to further analysis (A) Cell cycle distribution was analyzed by BrdU incorporation and DNA content flow cytometry detection. In the upper panel is shown a dual parameter dot plot of PI versus BrdU-Alexa 488. In the lower panel is shown a histogram display of DNA content versus counts. (B) Total protein extracts were fractionated by SDS-PAGE and immunoblotted with the indicated antibody.
(TIF)

Figure S8 **DDB1-depletd cells are a mix population of both ATM and RPA colocalizing foci cells and RPA only foci cells**. U2OS cells were transfected with control or siDDB1. Fixed cells were stained with the indicated antibodies. Nuclei were stained by DAPI. (A) A fluorescent picture of a representative microscopic field is shown. The white arrow indicates cells with both pATM and RPA signal. The yellow arrow indicates cells with RPA signal. (B) Cells with RPA only foci and RPA +pATM foci were counted and represented as bar graph. Mean value and error were calculated on three independent experiments. At least 50 cells per independent experiment were scored.
(TIF)

Figure S9 **Inactivation of either HLTF/RAD18 or CRL4^CDT2^ induces cell mortality.** (A) and (B) U2OS cells were transfected with the indicated siRNAs and both detached and adherent cells were harvested. Total protein lysates were analyzed by immunoblotting with the indicated antibodies. Δ indicates caspase3-cleaved PARP1 fragment. * indicates a background band. (C) HeLa cells were transfected with the indicated siRNAs. 48 Hrs after last trasfection, mortality was calculated according to the CytoTox kit manufacturer instruction. Cell mortality percentage increase over mock is shown in table. Data from 5 independent experiments are listed.
(TIF)
