# Supplementary figures and images for: Non-Canonical CRL4A/4BCDT2 Interacts with RAD18 to Modulate Post Replication Repair and Cell Survival
Source: PLoS One. 2013 Mar 29;8(3):e60000. doi: 10.1371/journal.pone.0060000 (PMC3612035; doi:10.1371/journal.pone.0060000)

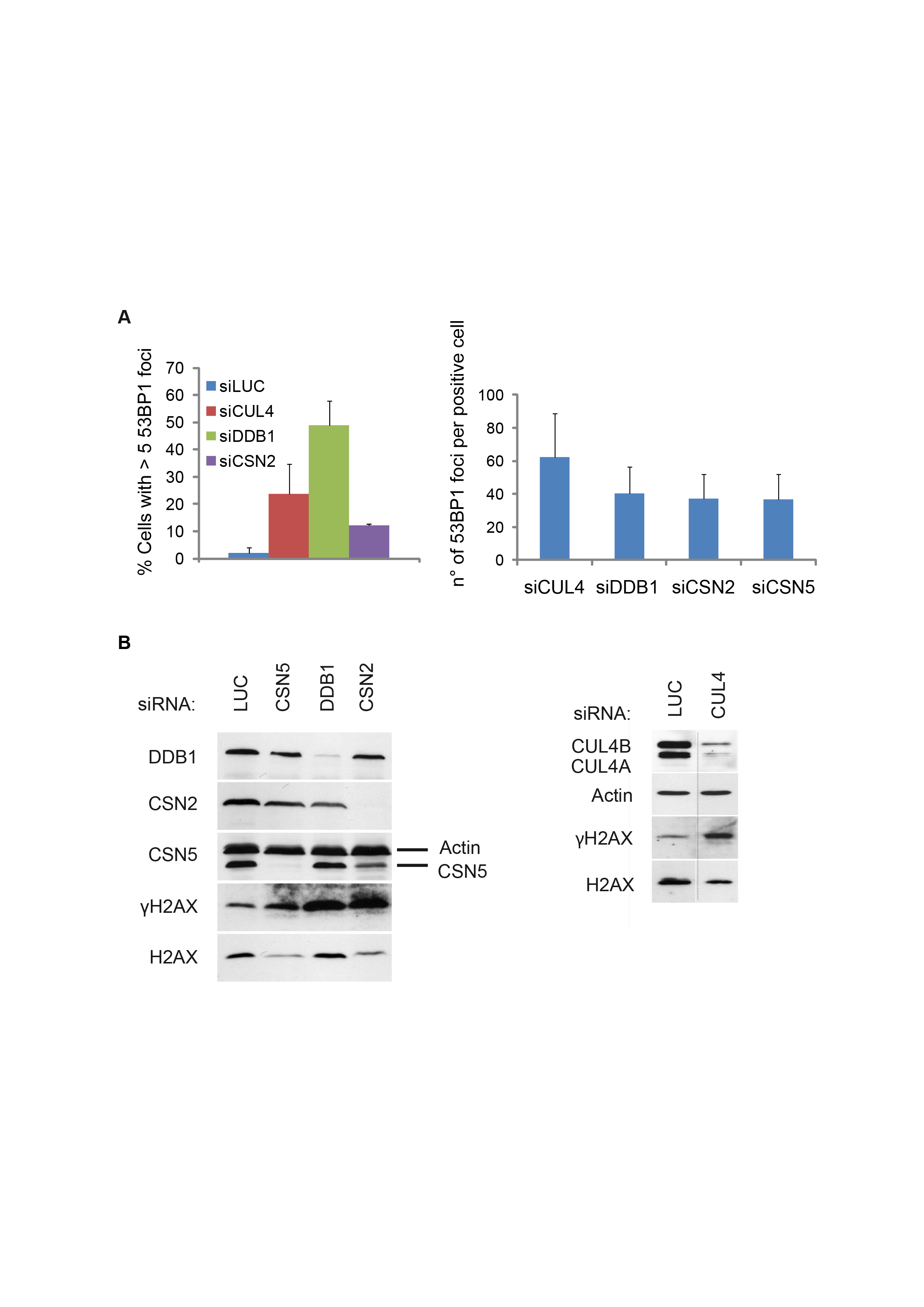

Supplement: Figure S1 — CSN-CRL4s depletion induces 53BP1 foci with different penetrance and severity and an increase in γH2AX. (A) HeLa cells were treated and analyzed by IF as in Figure 1A. For each independent experiment cells were scored as positive for 53BP1 foci if containing >5 foci. At least 50 cells per independent experiment were scored. Number of 53BP1 foci per cell were also counted. In the left panel it is graphically displayed the percentage of 53BP1 foci positive cells. In the right panel the mean of 53BP1 foci number per cell is graphically displayed as bar. Data represent mean +S.D. of three independent experiments. (B) Aliquots of the same HeLa cell populations in Figure 1A were harvested and cell lysates were resolved by SDS-PAGE and immunoblotted with the indicated antibodies. (TIF) [file pone.0060000.s001.tif]

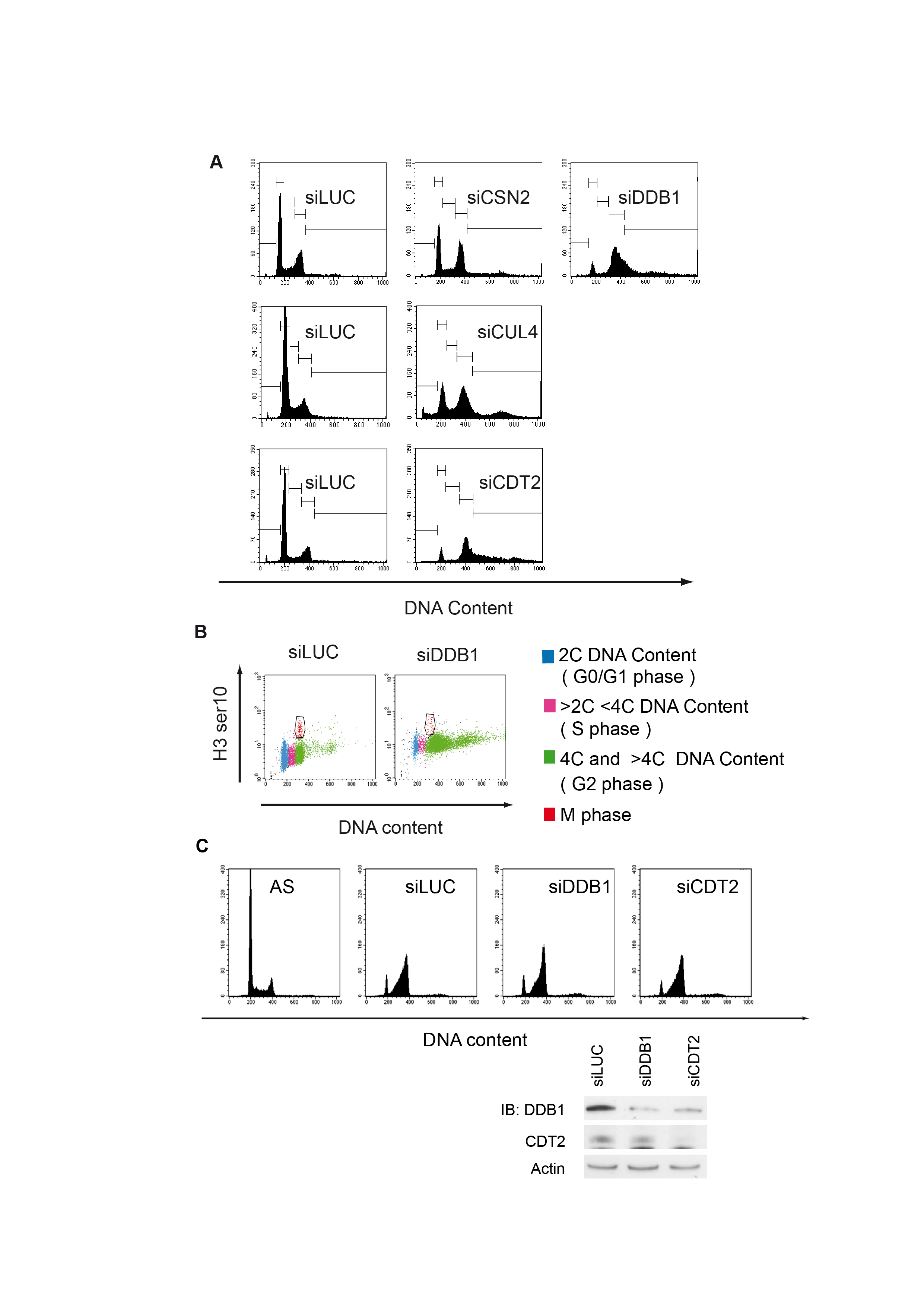

Supplement: Figure S2 — CSN-CRL4CDT2 depletion induces cell cycle block in G2 phase and re-replication. (A) HeLa cells were depleted of the indicated proteins and cell cycle distribution was analyzed by DNA content flow cytometry detection following propidium iodide staining. A representative FACS profile of many independent experiments with similar results is shown. (B) HeLa cells transfected with the indicate siRNAs were fixed and stained with anti–Ser10-phospho-H3 primary antibody, Alexa 488-conjugated secondary antibody, and PI. Cells were analyzed by FACS. The mitotic cells in square are positive for phospho-H3. (C) HeLa cells synchronized by DTB in mid S-phase and analyzed for IF in Figure 1C, were checked for cell cycle phase by FACS analysis and for protein depletion by immunoblotting. (TIF) [file pone.0060000.s002.tif]

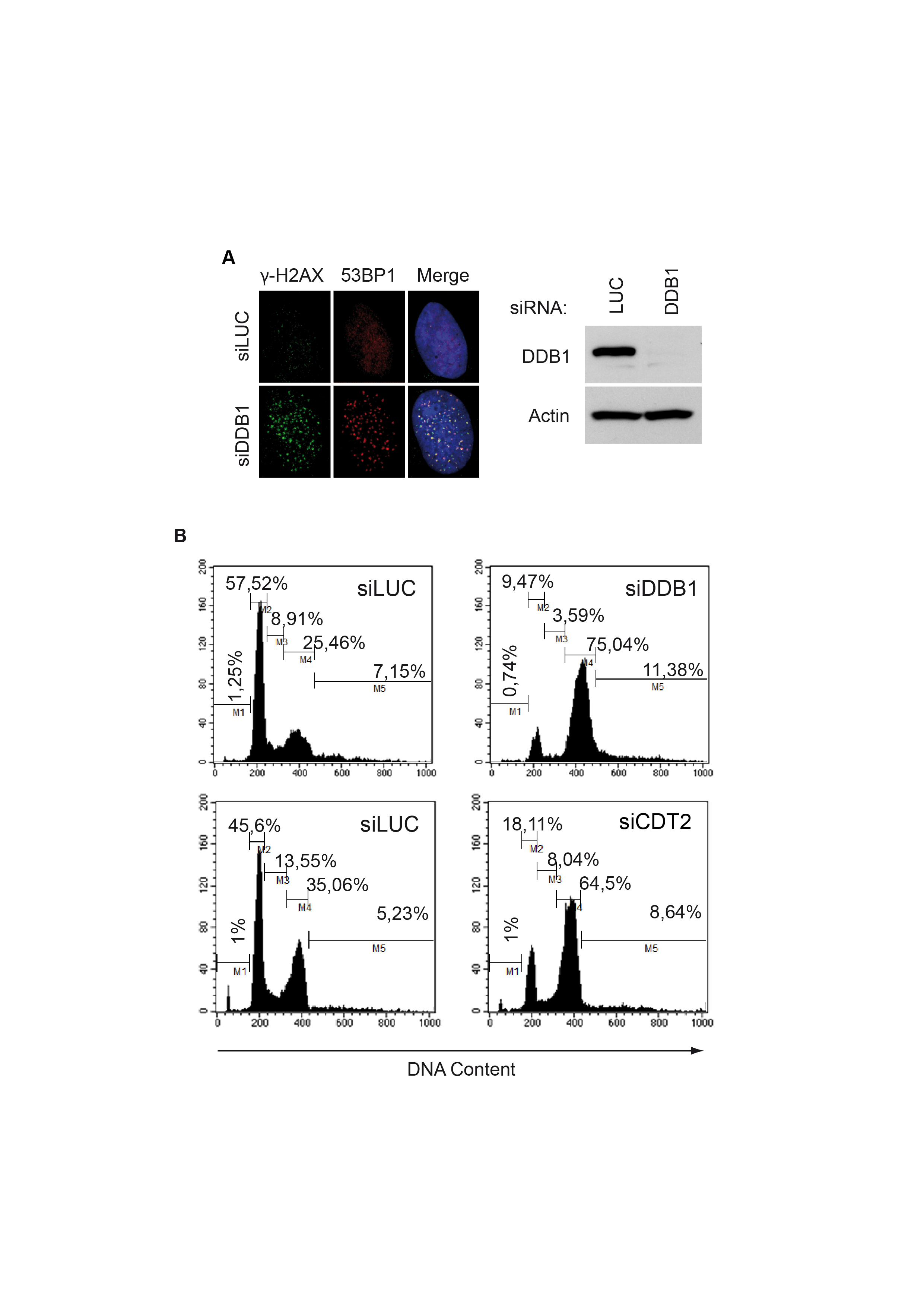

Supplement: Figure S3 — Either DDB1- or CDT2-depleted U2OS cells show DDR activation and cell cycle delay. U2OS cells depleted of the indicated proteins were harvested for further analysis. (A) Cells were fixed and stained with antibodies to H2AX phospho-S139 (γH2AX) and 53BP1; the nucleus was counterstained with DAPI. A fluorescent image of a representative nucleus is shown. A Cell sample was employed to check protein depletion by immunoblotting. (B) The cell cycle distribution was analyzed by DNA content flow cytometry detection following propidium iodide staining. A representative FACS profile with percentage of cells in each cell cycle phase of three independent experiments with similar results is shown. (TIF) [file pone.0060000.s003.tif]

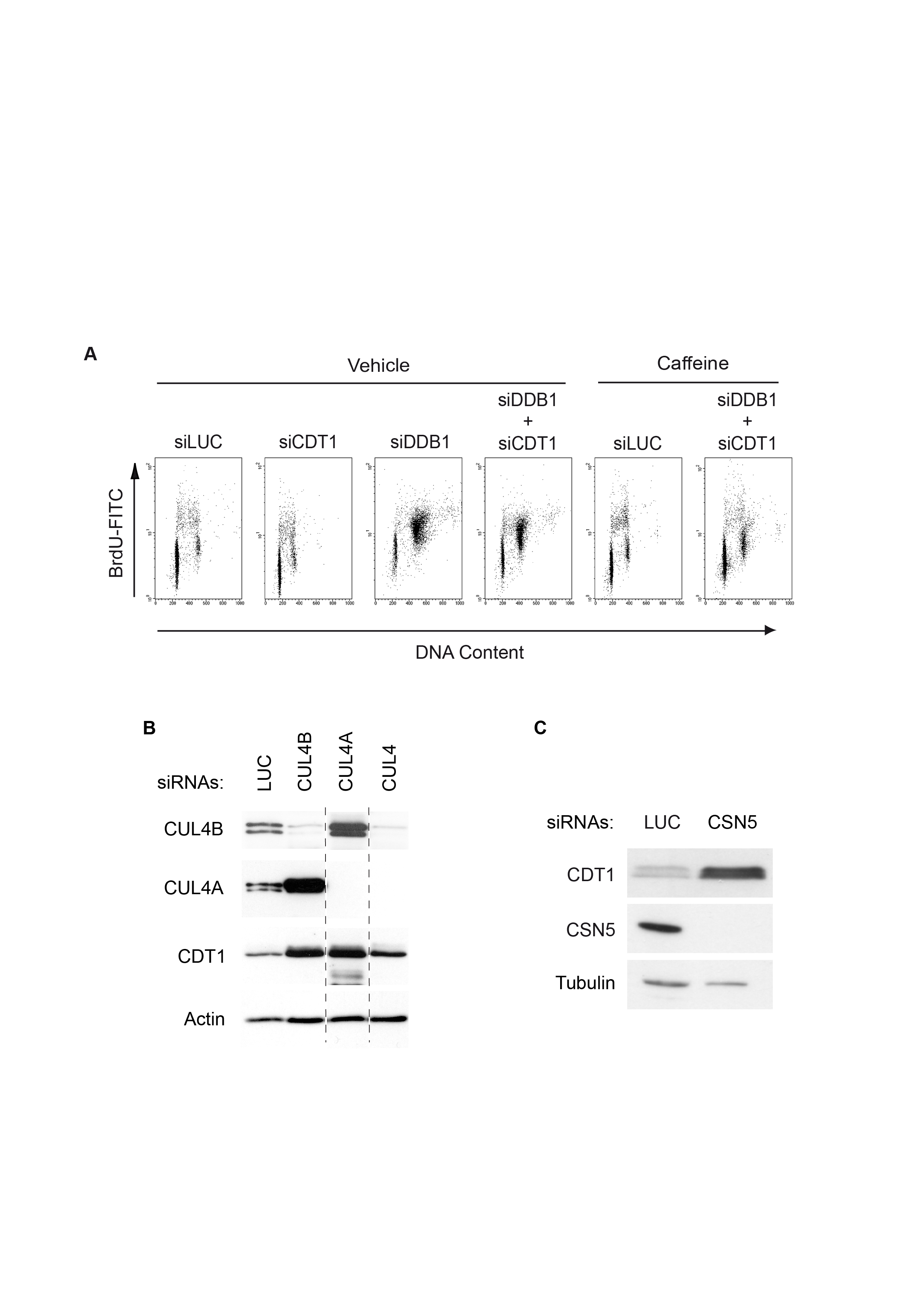

Supplement: Figure S4 — CSN-CRL4CDT2 has CDT1-dependent and CDT1-independent functions. (A) HeLa cells were transfected with the indicated siRNAs (± caffeine). Cell cycle distribution was analyzed by BrdU incorporation and DNA content flow cytometry detection. (B) 48 hrs following the last transfection cycle with control (siLUC), CUL4A (siCUL4A), CUL4B (siCUL4B) or both CUL4A and CUL4B (siCUL4) siRNAs, HeLa cells were harvested and processed for SDS-PAGE. Immunoblotting was performed with the indicated antibodies. (C) 48 hrs following the last transfection cycle with control (siLUC) and CSN5 (siCSN5) siRNAs, HeLa cells were harvested and processed for SDS-PAGE. Immunoblotting was performed with the indicated antibodies. (TIF) [file pone.0060000.s004.tif]

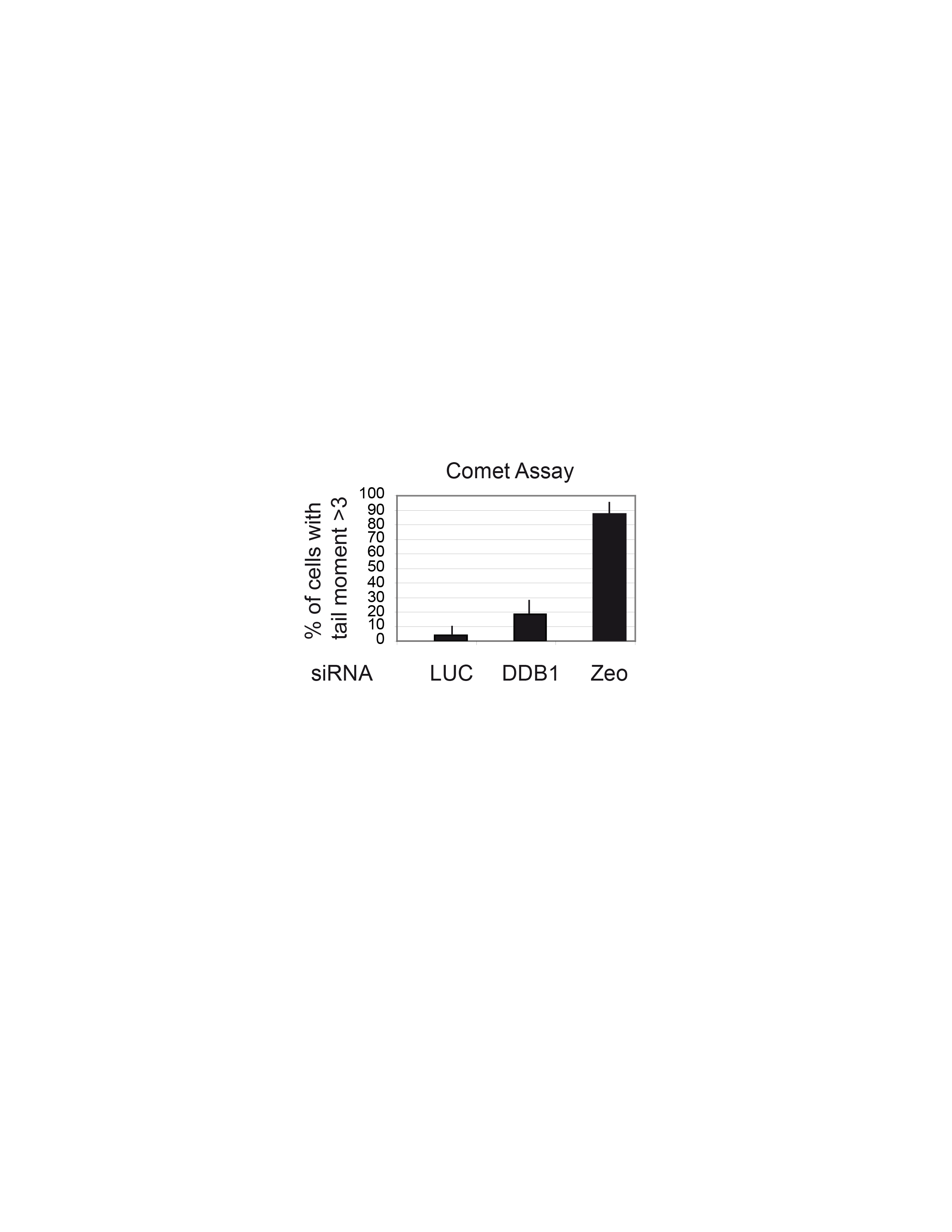

Supplement: Figure S5 — DDB1-depleted cells show DSBs. Alkaline comet assay on control and DDB1-depleted HeLa cells. A graphical representation of the mean percentage of cells with tail moment >3 as DNA damage parameter is shown. Mean value and error were calculated on three independent experiments. (TIF) [file pone.0060000.s005.tif]

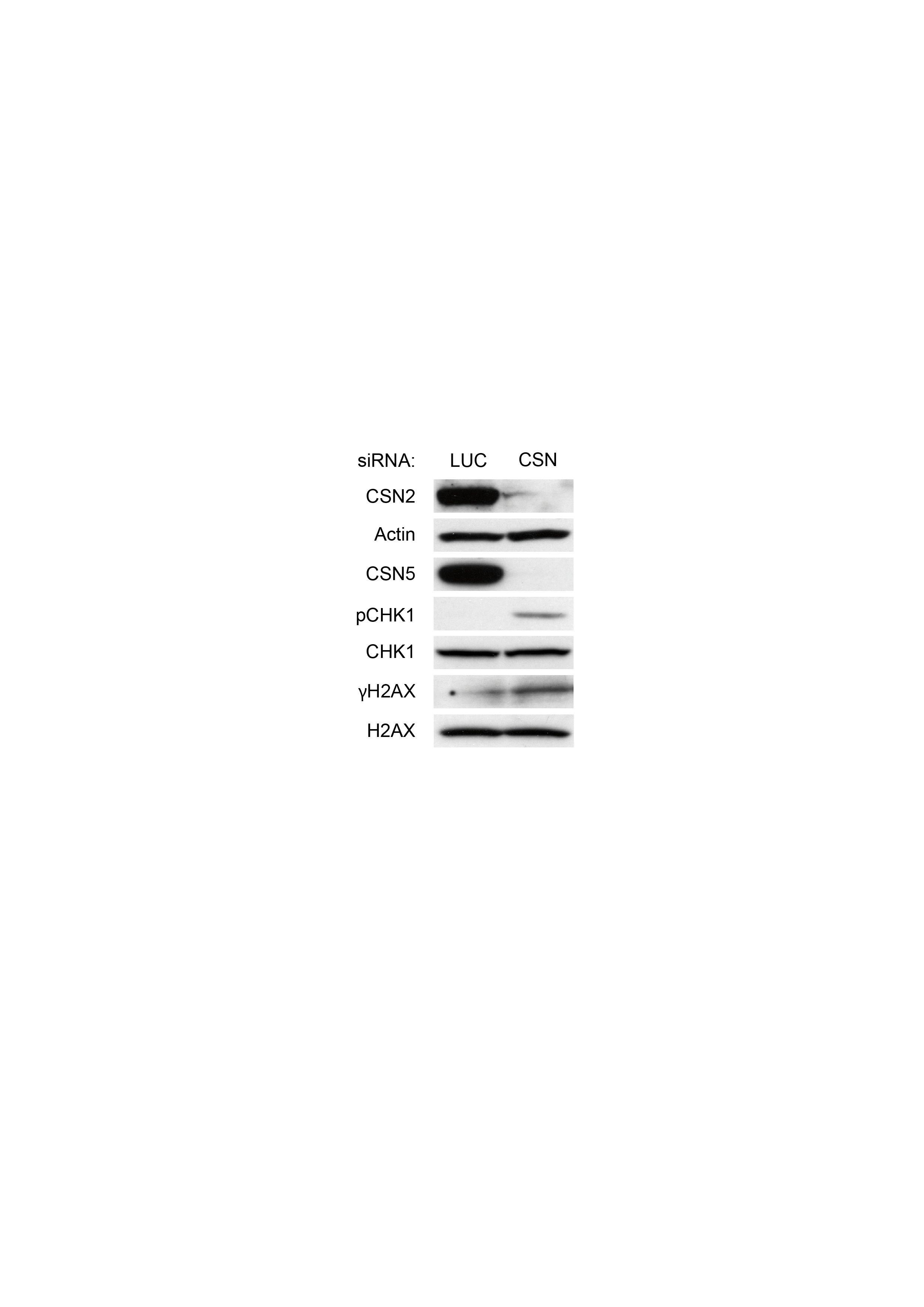

Supplement: Figure S6 — CSN depletion activates checkpoints. HeLa cells were harvested 48 hrs after the last transfection cycle with control (siLUC) or both CSN2 and CSN5 siRNA (siCSN). Total protein lysates were fractionated by SDS-PAGE and immunoblotted with the indicated antibody. (TIF) [file pone.0060000.s006.tif]

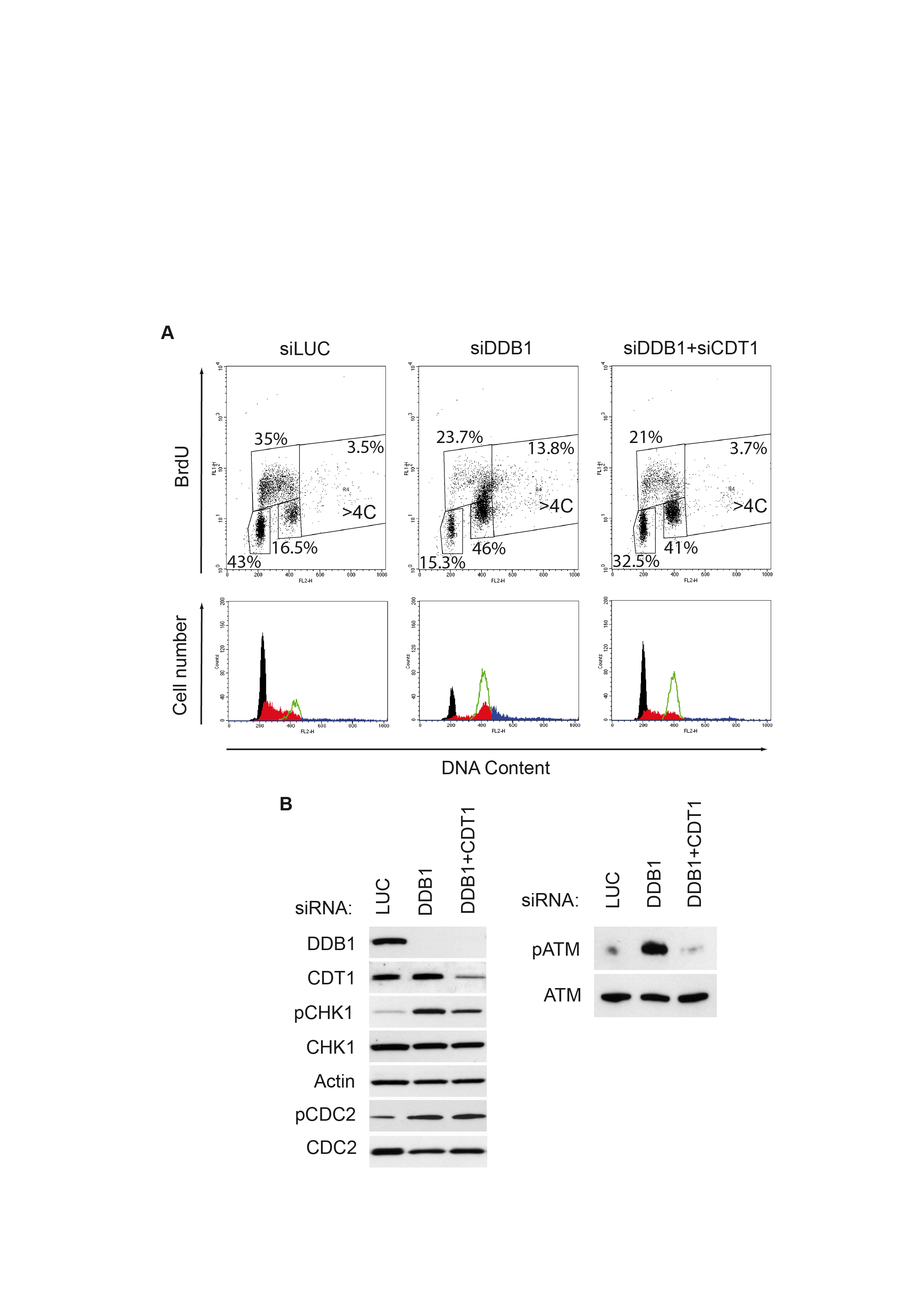

Supplement: Figure S7 — DDB1-depleted U2OS cells show a CDT1-dependent and a CDT1-independent delay in G2. U2OS cells were harvested 48 hrs after the last transfection cycle with control (siLUC), DDB1 (siDDB1) or both DDB1 and CDT1 (siDDB1+siCDT1) siRNAs and subjected to further analysis (A) Cell cycle distribution was analyzed by BrdU incorporation and DNA content flow cytometry detection. In the upper panel is shown a dual parameter dot plot of PI versus BrdU-Alexa 488. In the lower panel is shown a histogram display of DNA content versus counts. (B) Total protein extracts were fractionated by SDS-PAGE and immunoblotted with the indicated antibody. (TIF) [file pone.0060000.s007.tif]

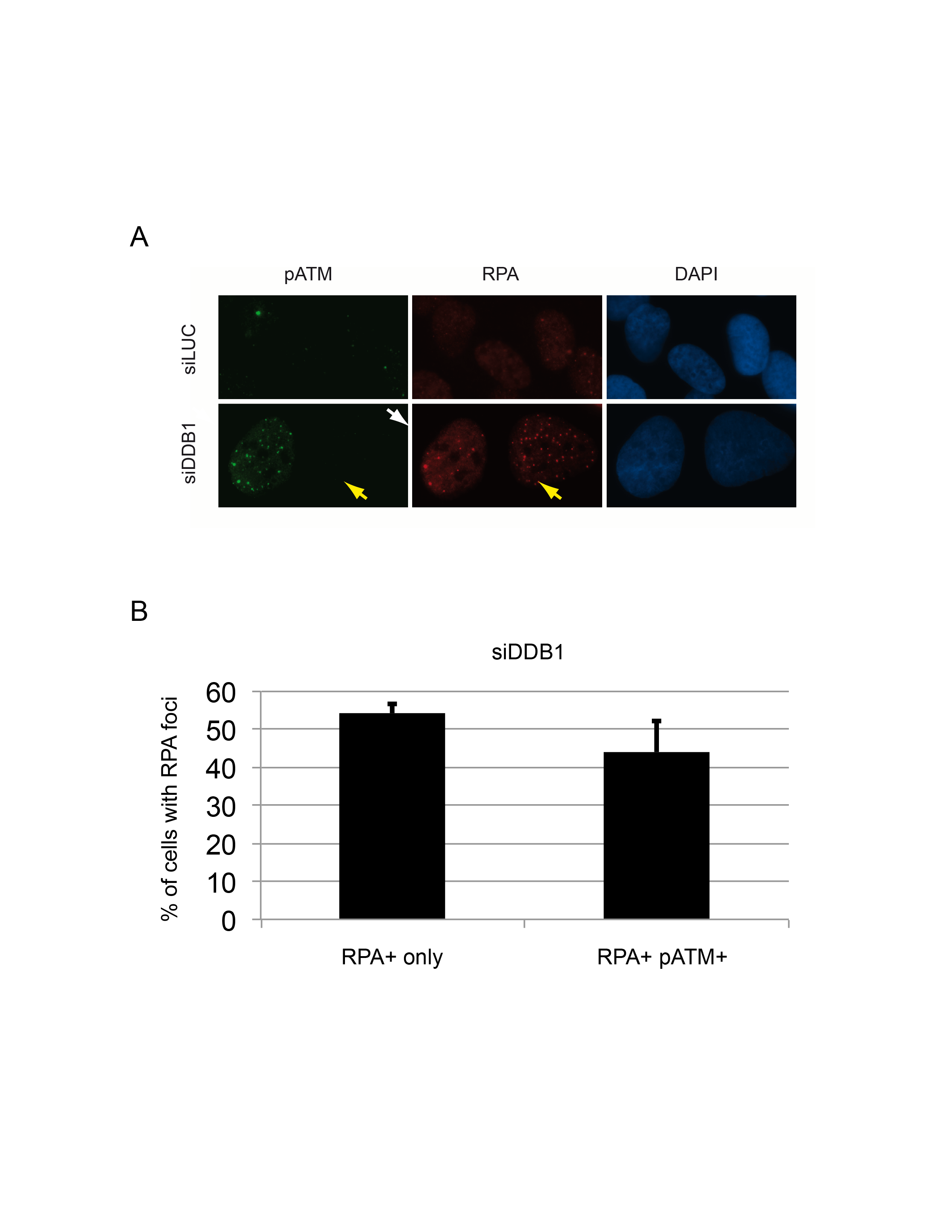

Supplement: Figure S8 — DDB1-depletd cells are a mix population of both ATM and RPA colocalizing foci cells and RPA only foci cells. U2OS cells were transfected with control or siDDB1. Fixed cells were stained with the indicated antibodies. Nuclei were stained by DAPI. (A) A fluorescent picture of a representative microscopic field is shown. The white arrow indicates cells with both pATM and RPA signal. The yellow arrow indicates cells with RPA signal. (B) Cells with RPA only foci and RPA +pATM foci were counted and represented as bar graph. Mean value and error were calculated on three independent experiments. At least 50 cells per independent experiment were scored. (TIF) [file pone.0060000.s008.tif]

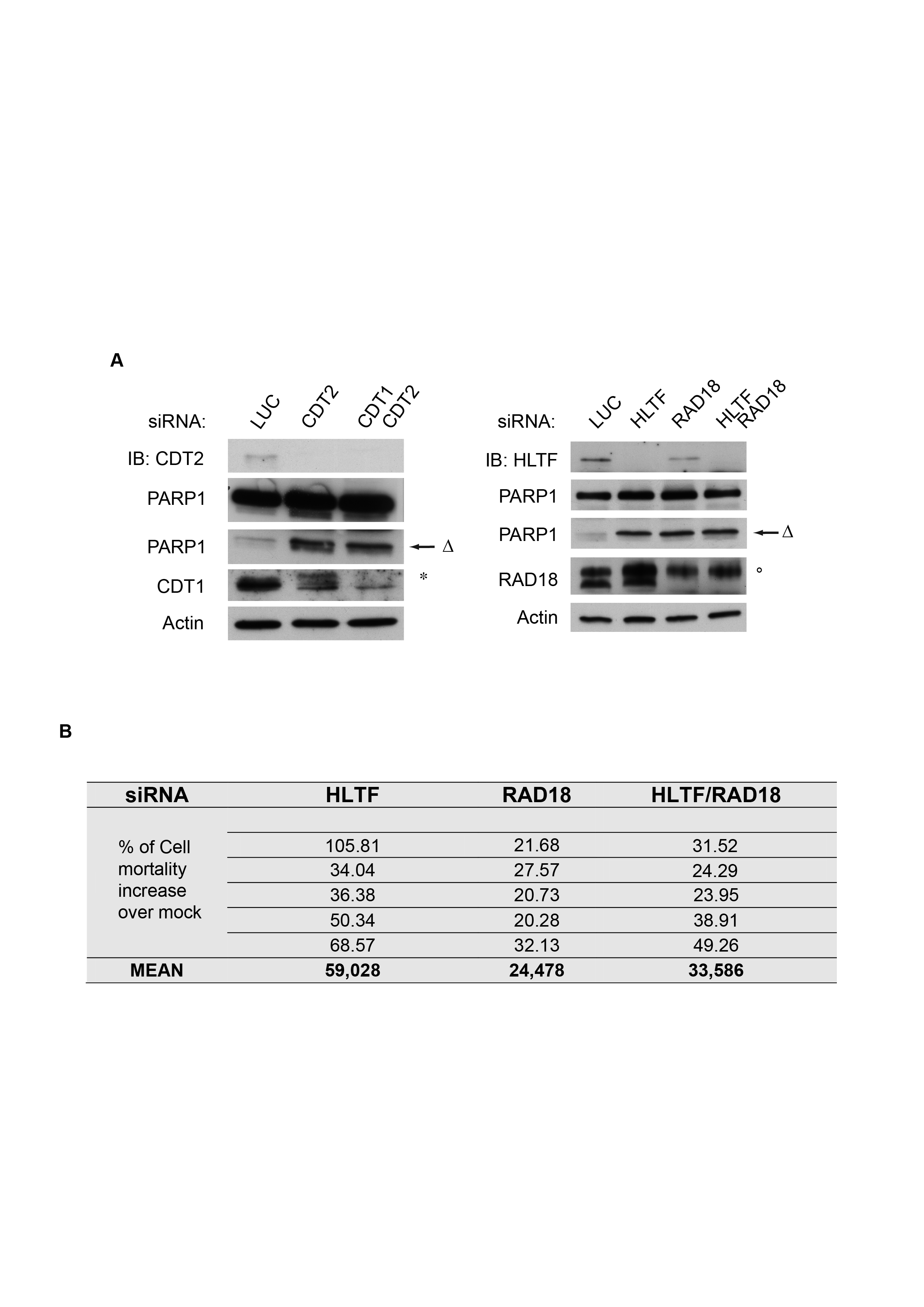

Supplement: Figure S9 — Inactivation of either HLTF/RAD18 or CRL4CDT2 induces cell mortality. (A) and (B) U2OS cells were transfected with the indicated siRNAs and both detached and adherent cells were harvested. Total protein lysates were analyzed by immunoblotting with the indicated antibodies. Δ indicates caspase3-cleaved PARP1 fragment. * indicates a background band. (C) HeLa cells were transfected with the indicated siRNAs. 48 Hrs after last trasfection, mortality was calculated according to the CytoTox kit manufacturer instruction. Cell mortality percentage increase over mock is shown in table. Data from 5 independent experiments are listed. (TIF) [file pone.0060000.s009.tif]
